# Associations between polygenic risk scores and accelerated brain ageing in smokers

**DOI:** 10.1017/S0033291723001812

**Published:** 2023-12

**Authors:** Zeyu Yang, Wei Zhao, Zeqiang Linli, Shuixia Guo, Jianfeng Feng

**Affiliations:** 1 MOE-LCSM, School of Mathematics and Statistics, Hunan Normal University, Changsha 410006, P.R.China; 2 Key Laboratory of Applied Statistics and Data Science, Hunan Normal University, College of Hunan Province, Changsha 410006, P.R.China; 3 School of Mathematics and Statistics, Guangdong University of Foreign Studies, Guangzhou, 510006, P.R.China; 4 Centre for Computational Systems Biology, Fudan University, Shanghai 200433, P.R.China; 5 Department of Computer Science, University of Warwick, Coventry CV4 7AL, England

**Keywords:** Ageing, brain age, enrichment analysis, polygenic risk score, smoking, sMRI

## Abstract

**Background:**

Smoking contributes to a variety of neurodegenerative diseases and neurobiological abnormalities, suggesting that smoking is associated with accelerated brain aging. However, the neurobiological mechanisms affected by smoking, and whether they are genetically influenced, remain to be investigated.

**Methods:**

Using structural magnetic resonance imaging data from the UK Biobank (*n* = 33 293), a brain age predictor was trained on non-smoking healthy groups and tested on smokers to obtain the BrainAge Gap (BAG). The cumulative effect of multiple common genetic variants associated with smoking was then calculated to acquire a polygenic risk score (PRS). The relationship between PRS, BAG, total gray matter volume (tGMV), and smoking parameters was explored and further genes included in the PRS were annotated to identify potential molecular mechanisms affected by smoking.

**Results:**

The BrainAge in smokers was predicted with very high accuracy (*r* = 0.725, MAE = 4.16). Smokers had a greater BAG (Cohen's *d* = 0.074, *p* < 0.0001) and higher PRS (Cohen's *d* = 0.63, *p* < 0.0001) than non-smokers. A higher PRS was associated with increased amount of smoking, mediated by BAG and tGMV. Several neurotransmitters and ion channel pathways were enriched in the group of smoking-related genes involved in addiction, brain synaptic plasticity, and some neurological disorders.

**Conclusion:**

By using a simplified single indicator of the entire brain (BAG) in combination with the PRS, this study highlights the greater BAG in smokers and its linkage with genes and smoking behavior, providing insight into the neurobiological underpinnings and potential features of smoking-related aging.

## Introduction

Smoking has become one of the greatest threats to world health, with approximately eight million people die from smoking each year. Smoking accelerates the aging process of organs and leads to a variety of diseases, such as circulatory and respiratory diseases (Wu et al., 2019). In addition, smoking may lead to a variety of neurodegenerative diseases and neurobiological abnormalities, such as cognitive decline and dementia, suggesting that smoking is associated with accelerated brain ageing (Vňuková, Ptáček, Raboch, & Stefano, 2017). Although many previous studies have reported widespread abnormalities of brain structure and function in smokers (Elbejjani et al., 2019; Lin et al., 2020; Yang, Zhang, Cheng, & Zheng, 2020), the neurobiological mechanisms with smoking remain to be elucidated. Hence, defining novel phenotypes capturing global age-related changes in brain could, via variation in the genome and changes in the brain that associate with these aberrancies, provide novel biological insights.

Recently, a ‘brain age estimation’ paradigm based on neuroimaging, particularly structural magnetic resonance imaging (sMRI), has been successfully used to detect delayed brain ageing in healthy and clinical populations (Franke & Gaser, 2019). The method uses the BrainAge Gap (BAG), that is the difference between estimated and chronological brain age, to measure the deviation of the brain from healthy ageing in clinical populations. Our previous study has quantified the association between smoking parameters and BAG, including smoking status, amount of smoking, and smoking cessation (Linli, Feng, Zhao, & Guo, 2022). In addition, BAG has been confirmed to be heritable (Cole et al., 2017) and has polygenic overlaps with brain disorders such as schizophrenia (SCZ), bipolar disorder, multiple sclerosis, and Alzheimer's disease with BAG (Luders, Cherbuin, & Gaser, 2016). If smokers exhibit macroscopic BAG, then this may be associated with genetic variants (Jonsson et al., 2019). Elucidating this relationship would enhance our understanding of the molecular genetic basis of structural brain abnormalities in smokers.

Given the small effect of a single genetic variant on susceptibility to complex diseases, the polygenic risk score (PRS) was developed to measure the additive effects of multiple single nucleotide polymorphisms (SNPs) based on large-scale genome-wide association studies (GWAS) summary statistics and has been successfully applied in psychiatric disorders (Purcell et al., 2009; Ranlund et al., 2018; Torkamani, Wineinger, & Topol, 2018). One potential application of the PRS is in the field of imaging genetics, which offers the opportunity to investigate genetic factors that may affect brain structure and function (Arslan, 2018; Mufford et al., 2017). Functional annotation of genes included in PRS calculation to explore potential molecular mechanisms can improve the understanding of abnormal physiological activity or disease etiology. And PRS has been shown to be a useful strategy for assessing the role of polygenic risk in smoking (Kim et al., 2021; Ohi et al., 2020; Vink et al., 2014). However the relationship between smoking-related PRS and BAG has not explored.

This study aims to investigate the neurobiological mechanisms behind smoking by examining the relationship between PRS and BAG in smokers in a middle-aged and elderly population, using data from UK Biobank, the largest neuroimaging database. We assume that (i) both BAG and PRS are valuable features in smoking, (ii) smokers have positive BAG and higher PRS, and (iii) BAG is significantly associated with PRS, which is mediated by the amount of smoking and tGMV and related to specific molecular mechanisms (e.g. neurotransmitter synaptic and ion pathway activity).

## Materials and methods

### Participants

The study included structural MRI examinations of 33 293 middle-aged and older people (44 to 81 years, 18 626 smokers, and 14 667 non-smokers) from the UK Biobank. The UK Biobank obtained ethical approval from the Research Ethics Committee (RECreference 11/NW/0382) and got written informed consent from each subject. Data access was granted in accordance with UKB application 19 542 (PI Jianfeng Feng). The detailed exclusion criteria were presented in our previous paper (Linli et al., 2022).

### Imaging data collection and preprocessing

The UK Biobank used a standard Siemens Skyra 32-channel 3 T scanner (Siemens Medical Solutions, Germany) for whole brain MRI with a resolution of 1 × 1 × 1 and a view field of 208 × 256 × 256. The details of the image acquisition are provided on the UK Biobank website in the form of a protocol (http:// biobank.ctsu.ox.ac.uk/crystal/refer. cgi?id=2367).

All UK Biobank sMRI data were preprocessed in the CAT12 toolbox with default settings. The detailed preprocessing steps can be referred to our previous paper (Linli et al., 2022). Automated anatomical labeling 3 (AAL3) atlas (Rolls, Huang, Lin, Feng, & Joliot, 2020) was used to divide the brain into 166 regions (online Supplementary Table S4) of interest and obtain regional gray matter volumes (rGMV). Finally, rGMV, tGMV and estimated total intracranial volume (TIV) were obtained for each subject, with tGMV was calculated as the sum of all 166 rGMV and TIV was calculated as the sum of gray matter, white matter, and cerebrospinal fluid volumes in natural space.

### Genetic data preprocessing (genotyping)

Genomic DNA was extracted from whole blood using standard protocols. All individuals were genotyped on the Illumina Global Screening Array-24v1.0BeadChip. The chip provides data for 642 824 fixed gene variants and 53 411 customized variants. SNPs with minor allele frequencies <1%, call rates <95%, Hardy–Weinberg equilibrium *p* < 10^−5^ were excluded from analysis, and individuals with excessive missingness >5% and sex mismatches >0.90 were excluded from the study (Wang, Lu, Yu, Gibbs, & Yu, 2013). As not all subjects were collected for genetic data, 616 339 SNPs on 23 845 subjects (11 358 males, 12 487 females) were eventually included in final genetic sample.

### Analysis overview

**Figure 1.**
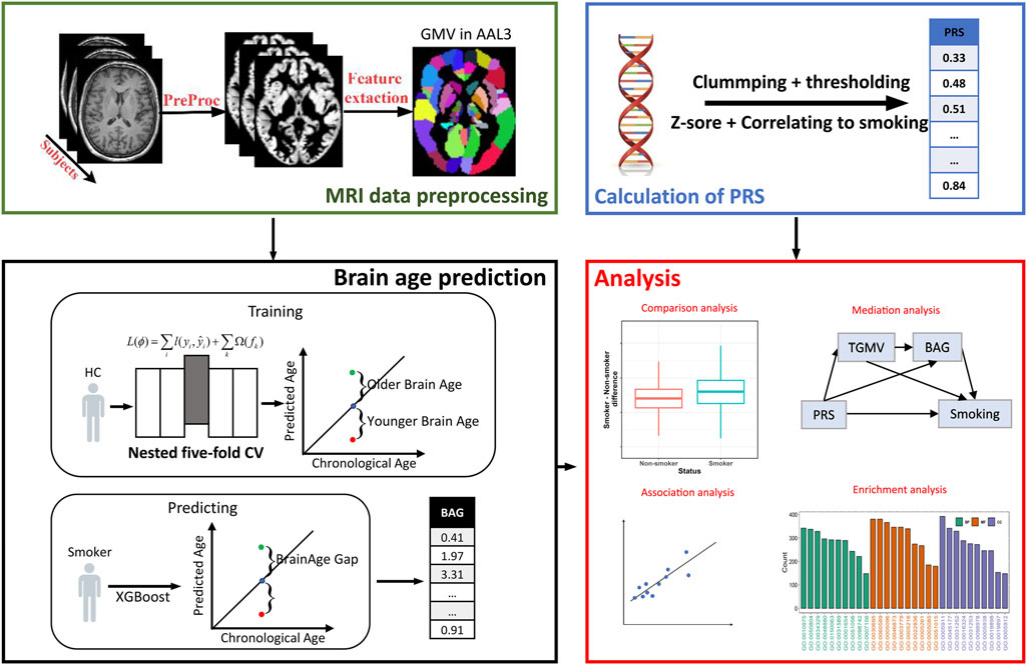
Flow diagram of the analysis approach used in the study. **Green box:** The gray matter of images is segmented after the common preprocessing procedure and partitioned into 166 regions of interest based on the automated anatomical labeling 3 (AAL3) atlas, which further are residualized for sex, ethnicity, handedness, BMI, scanning site, alcohol consumption, and TIV using linear regression models and then input to orange box. **Black box:** Subjects are split into smokers and never-smoking controls. Controls are trained on XGBoost predictors using a nested five-fold CV framework. The final five XGBoost predictors with optimal parameters are used to predict the brain age of smokers. **Blue Box:** Calculating PRS from DNA data by clumping to eliminating linkage disequilibrium effects and thresholding to select the relevant genetic variants. Then PRS most related to smoking were chosen for the following analyses after Z-score. **Red Box:** Statistical analysis in the study includes comparative analysis (BrainAge Gap or PRS between smoker and control), association analysis (with continuous smoking parameter, tGMV and PRS), mediation analysis and enrichment analysis for gene included in PRS.

Figure 1 outlines the analytical pipeline used in this study. After preprocessing, all 166 rGMVs were residualized for sex, ethnicity, handedness, BMI, alcohol consumption, TIV, scanning site and education using linear regression models. Then, the PRS and BAG were calculated as described in the following sections. Finally, the optimal PRS and corrected brain age was used in subsequent analyses, including association analysis, mediation analysis, comparative analysis and enrichment analysis.

### BAG prediction

The 166 rGMV were used as features to build a brain age prediction model based on the extreme Gradient Boosting (XGBoost) algorithm (de Lange et al., 2020; Kaufmann et al., 2019; Linli et al., 2022), and the predicted brain age was then used to obtain an unbiased predicted brain age (Beheshti, Nugent, Potvin, & Duchesne, 2019; Le et al., 2018; Liang, Zhang, & Niu, 2019; Smith, Vidaurre, Alfaro-Almagro, Nichols, & Miller, 2019). The BAG was defined as the gap between the unbiased predicted brain age and chronological age, which characterizes the individual age-related accelerated or decelerated brain ageing. Finally, comparison on BAG was performed to check whether smoking is associated with BAG. The detailed calculation of BAG can be referred to Supplemental Materials.

### Calculation of smoking-related PRS

A threshold approach was used to calculate a PRS to assess the cumulative genetic risk effect of smoking (Santoro et al., 2018). The steps are as follows:

1. The large-scale GWAS results from the Data Repository for University of Minnesota (Liu et al., 2019) were used as a discovery sample.
2. P-value-informed clumping with a cutoff of *r^2^* = 0.1 in a 250-kb window was performed.
3. A *p*-value threshold (*P_T_*) was used for the selection of the SNPs. Identification of risk alleles for SNPs under the *P*_*T*_ threshold (*P* < *P*_*T*_).
4. Using the sample recruited for this study as the target sample, a PRS was calculated in the target sample according to the equation in Supplementary Methods.

To reduce bias due to artificial selection of individual thresholds, since the optimal *p*-value threshold is unknown a priori, we calculated PRSs for 100 *P*_*T*_ thresholds (*P*_*T*_ value range is between [0.005,0.5], in steps of 0.005). The top 10 PRS with the highest partial correlation coefficient with smoking were selected (Kang et al., 2022) with covariates including age, sex, handedness, BMI, alcohol status, TIV and the first five components generated by the population stratification of PLINK 1.9 (Purcell et al., 2007). The z-score of the optimal PRS obtained at each *P_T_* threshold was standardized for subsequent calculations.

Discovery and target samples in PRS calculation should not be overlapped, which can inflate the significance. In this article, all target samples and part of the discovery samples are from the UK biobank, so we remove the duplicated samples from discovery samples. Besides, there are five summary statistics in the GSCAN publication, and each of them was used in PRS calculations. Smoking Initiation summary statistics were used as our discovery samples, as we found that PRS had the greatest correlation with smoking in the GSCAN publication's Smoking Initiation Summary Statistics.

### Statistical analysis

#### Association analysis

A generalized linear model was used to investigate the association between the PRS (PRS under the optimal threshold *P_T_*) and BAG, tGMV, and smoking parameter (Pack-year & quitting duration), with PRS as the independent variable and BAG, tGMV, and smoking parameter as the dependent variables respectively. Furthermore, the associations between PRS and rGMVs were investigated respectively to determine which brain regions were strongly associated with PRS. Only significant brain regions survived under FDR correction were shown in the result. Note that sex, age, handedness, BMI, alcohol status, TIV, site and education were used as the covariates for these two kinds of association analysis.

#### Mediation analysis

Mediation analysis was performed using the R package mediation to test the hypotheses of (1) whether the relationships between the PRS (independent: X) and amount of smoking (dependent: Y) were mediated through BAG (mediator: M), (2) whether the relationships between the PRS (X) and BAG (Y) were mediated through amount of smoking (M), and (3) whether tGMV and BAG or pack-year simultaneously as mediating variables in the above two hypotheses. The significance of the mediators was estimated by the bias-corrected bootstrap method (1000 random samplings). Confounding variables as in the association analysis were regressed out in the mediation model. The percentage of the mediation effect (PM) that could be explained by the mediator (indirect effect) was measured using the formula: 100% × (τ−τ’)/(τ). Detailed descriptions of the mediation analyses can be found in the Supplementary Material.

#### Comparative analysis

A two-sample *t* test was used to test for the differences in BAG and PRS between the smoking and non-smoking groups. Furthermore, the differences in BAG and PRS between the smoking subgroups and the non-smoker group were examined to test whether smoking status was significantly associated with the BAG and PRS. Cohen's d statistics was used to measure the effect size of the difference between groups.

### Enrichment analysis

SNPs included in the PRS calculations were annotated to find their corresponding genes using the ensembl website (asia.ensembl.org/). These genes were enriched using the ‘clusterProfiler’ R package to find the enrichment of the target genes in the Gene Ontology (GO) term and the Kyoto Encyclopedia of Genes and Genomes (KEGG) pathway. The functional properties of these genes are characterized by GO and KEGG terms, with GO terms including molecular function (MF), cellular component (CC), and biological process (BP). Adjusted *p*-values were obtained using the FDR method.

## Results

### Demographics

Of the 33 293 participants, the smoker was 55.95% (*N* = 18 626, male/female = 9233/9393) with mean age of 64.20 ± 7.51 and the non-smoker was 44.05% (*N* = 14 667, male/female = 8249/6418) with mean age of 63.12 ± 7.52. Among smokers, the proportion of males is 49.6%, while among non-smokers, the proportion of males is 43.8%. There were no significant differences in handness between two groups. Detailed characteristics of these participants were provided in online Supplementary Table S1.

### Prediction performance of XGBoost predictor for brain age

The brain age prediction accuracy of the XGBoost model on the non-smoker group was *r* = 0.712, CI = [0.703, 0.719], RMSE = 5.280, MAE = 4.220, based on a nested five-fold CV framework. BAG for the non-smokers was negatively correlated with chronological age (*r* = −0.707), as shown in online Supplementary Figure S1. The corrected BrainAge for the non-smokers correlated more strongly with chronological age (*r* = 0.896, CI = 0.893–0.899, RMSE = 3.722, MAE = 2.997) and the corrected BAG was orthogonal to chronological age (*r* ≈ 0) (online Supplementary Figure S1).

**Figure 2.**
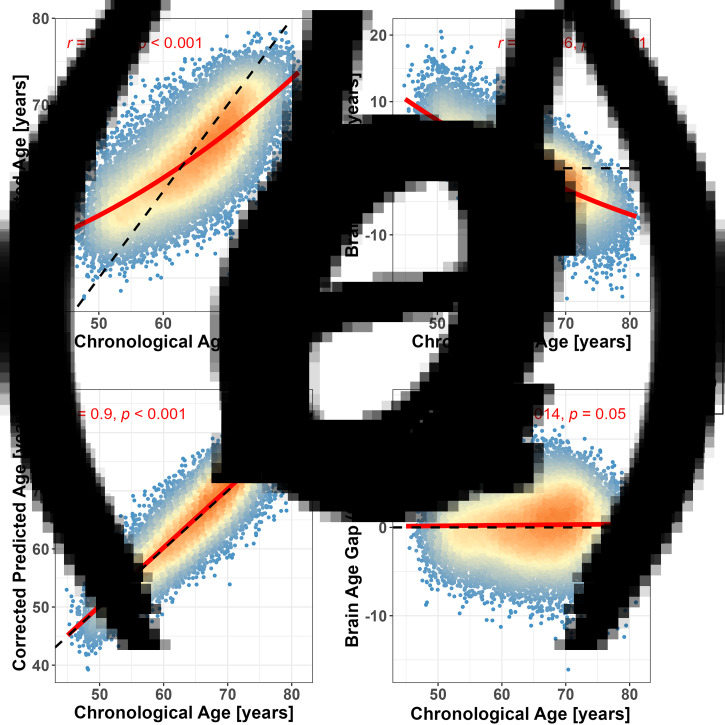
Prediction performance of the XGBoost predictor for brain age (in smoker group) and comparison between groups. **(a)** Correlation between the BrainAge (i.e. predicted age) and the chronological age with *r* = 0.725, *p* < 0.001. **(b)** Correlation between the BrainAge Gap and the chronological age with *r* = − 0.706, *p* < 0.001. (A) and (B) show that the brain age is overestimated in younger subjects and underestimated in older subjects. **(c)** and **(d)** show the correlation between corrected the BrainAge (*r* = 0.9, *p* < 0.001) and the BrainAge Gap (*r* = 0.014, *p* = 0.05) and the chronological age after bias adjustment. The slope of the black dotted line in A and C is 1, while that in B and D is 0. The red line in A and C is the fitted curve with the linear and quadratic representations of the chronological age, while that in B and D is a fitted curve with the linear effect of the chronological age.

The brain age predictor trained from the non-smokers was used to predict the brain age of the smoking group. The correlation between predicted and chronological age in the smoking group was *r* = 0.725, CI = [0.718, 0.732], RMSE = 5.179 and MAE = 4.16 (Fig. 2a). Similarly, BAG was also correlated with chronological age (*r* = −0.706, Fig. 2b). Using the correlation coefficients of the model from the non-smokers to remove the BrainAge bias of smokers, the corrected BrainAge of the smoker group was more relevant to chronological age than that before (*r* = 0.900, CI = [0.898, 0.903]; Figure 2c), and the corrected BAG was almost orthogonal to the chronological age (*r* = 0.014, *p* = 0.05; Figure 2d).

**Figure 3.**
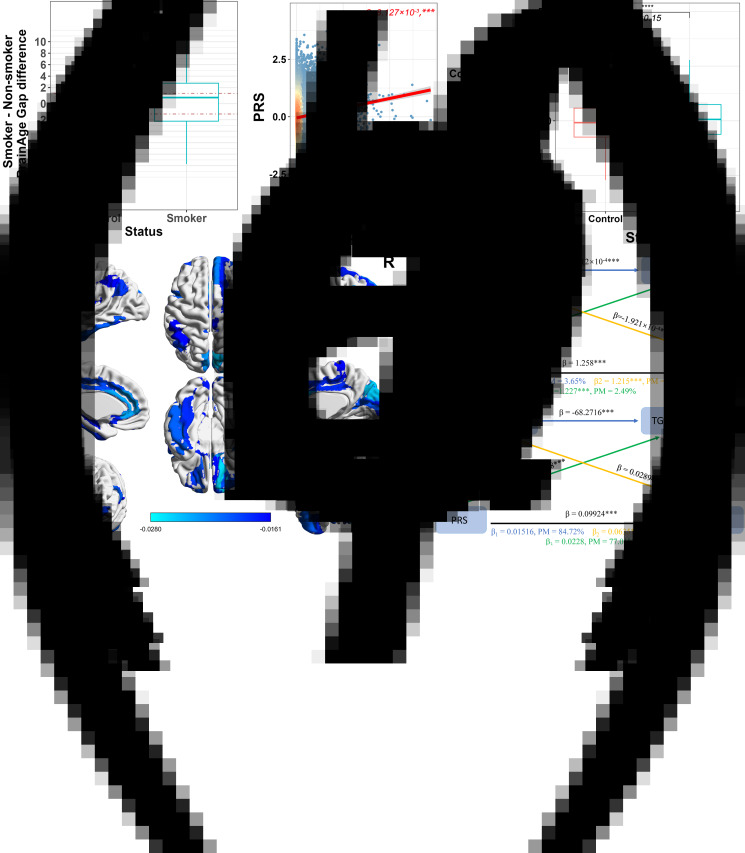
The results of correlation and comparison analysis about PRS and mediation analysis. **(a)** Difference between smoker group and non-smokers in the corrected BrainAge Gap. **(b)** Association between the PRS and Pack.year in smoker group. **(c)** Difference between smoker group and controls in the PRS. **(d)** The significant (adjusted *p* < 0.001) correlation coefficient between PRS and GMV of each brain region. **(e)** Mediation analysis results. Yellow line: Mediation by tGMV of the association between PRS and Pack.year. Green line: Mediation by BAG of the association between PRS and Pack.year. Blue line: Mediation by tGMV and BAG of the association between PRS and Pack.year. **(f)** Mediation analysis results. Yellow line: Mediation by Pack.year of the association between PRS and BAG. Green line: Mediation by tGMV of the association between PRS and BAG. Blue line: Mediation by Pack.year and tGMV of the association between PRS and BAG. (****, *p* < 0.0001, ***, *p* < 0.001, **, *p* < 0.01, *, *p* < 0.5, NS, non-significant).

As shown in Fig. 3a, smokers had a bigger BAG than non-smokers (Cohen's *d* = 0.074, *p* < 0.0001). After further subgrouping of the smoking group according to severity of smoking, individuals with the highest severity (smokers who are currently smoking and have a high amount) had the largest difference in mean BAG compared with the non-smokers (Cohen's *d* = 0.307, *p* < 0.001), with the difference decreased with the severity of smoking (online Supplementary Figure S3).

### Optimal PRS selection in relation to smoking

Correlations between 100 sets of PRS and smoking were calculated and the 10 most strongly correlated sets of PRS were obtained, which had *P_T_* of 0.04, 0.05, 0.045, 0.055, 0.13, 0.025, 0.12, 0.19, 0.2 and 0.115 (online Supplementary Table S6), with correlation coefficients and significance levels as shown in online Supplementary Table S6 of the supplementary material (subsequent analysis show only *P_T_* for 0.04, Fig. 3b, with similar results at other *P_T_* thresholds).

The smoking group (Smoker) had a higher PRS compared to non-smokers (Cohen's *d* = 0.15, *p* < 0.0001, Fig. 3c), and when the smoking group was further divided, all smoking subgroups had significantly different PRS from the non-smoking group (Cohen 's *d* = [0.005,0.383], online Supplementary Figure S4), with differences between smoking subgroups and non-smokers decreasing with the degree of smoking status, with the current smoker group having the largest mean PRS and the largest difference from the non-smoking group (Cohen's *d* = 0.383, *p* < 0.0001, online Supplementary Figure S4), with the other smoker groups possessing the smallest differences.

### Association analysis of PRS with BAG and tGMV

As shown in Table 1, we found a positive correlation between PRS and BAG (*β* = 0.08, *p* = 3.09 × 10^−4^). In contrast, tGMV was significantly negatively correlated with PRS (*β* = −171.28, *p* = 5.28 × 10^−4^) and duration of quitting was significantly negatively correlated with PRS (*β* = −0.75, *p* = 3.06 × 10^−5^); the correlation between PRS and either BAG, tGMV or duration of quitting was slightly attenuated (*β* = 0.04, *p* = 0.424; *β* = −146.45, *p* = 0.0142; *β* = −0.36, *p* = 0.0183), while after controlling the effect of Pack-years. This means that a higher risk at the genetic level might imply a more severe smoking habit (greater smoking, more difficulty in quitting), which might suggest that the genes we looked for might have some relationship with the level of smoking addiction, and therefore enrichment analysis of these genes is warranted.

**Table 1.** Association of PRS (P_T_ = 0.04) with BAG, tGMV and smoking parameter

Group	Model1			Model2		
	*β*(SE)	*t*-value	*p*-value	*β*(SE)	*t*-value	*p*-value
	BAG					
Smokers (unadjusted Pack.year)	0.09(0.032)	2.824	0.0048	0.08(0.032)	2.708	0.0068
Smokers (adjust Pack.year)	0.04(0.049)	0.895	0.371	0.04(0.049)	0.800	0.424
Smokers and non-smokers	0.08(0.024)	3.607	3.10 × 10^−4^	0.08(0.024)	3.608	3.09 × 10^−4^
	tGMV					
Smokers and non-smokers (unadjusted Pack.year)	−364.24(74.283)	−4.904	9.49 × 10^−7^	−171.28(49.408)	−3.467	5.28 × 10^−4^
Smokers and non-smokers (adjusted Pack.year)	−273.56(89.784)	−3.047	0.00232	−146.45(59.736)	−2.452	0.0142
	Quitting duration					
Unadjusted Pack.year	−0.76(0.179)	−4.226	2.42 × 10^−5^	−0.75(0.179)	−4.172	3.06 × 10^−5^
Adjusting Pack.year	−0.31(0.151)	−2.071	0.0384	−0.36(0.151)	−2.359	0.0183

Note: In this association analysis, the predictor variable was PRS and response variables were shown in the table. Model 1: Adjusted for sex, age. Model 2: Adjusted for sex, age, TIV, handedness, BMI, alcohol status, site, education.

At the brain region level, the results of the correlation analysis between PRS and rGMV are shown in Fig. 3d (online Supplementary Table S7), with the most strongly correlated (largest Pearson correlation coefficient) brain regions being: Thalamus (Reuniens and Anteroventral Nucleus), IFG pars orbitalis, Middle frontal gyrus, Lateral orbital gyrus, Amygdala, Superior frontal gyrus (medial orbital), Superior temporal gyrus and Anterior cingulate cortex (pregenual). Several of these brain areas, including the Superior frontal gyrus, Middle frontal gyrus and Anterior cingulate cortex, are involved in the reward circuit, or what is known as the mesolimbic dopamine system.

### Results of mediation analysis

Firstly, we consider the relationship between PRS and Pack-year, with BAG and tGMV as mediation variables. Figure 3e shown that both BAG and tGMV had a partial mediating effect (*β* = 1.227, *p* < 0.001, PM = 2.49% for BAG; *β* = 1.215, *p* < 0.001, PM = 3.34% for tGMV). When considering both BAG and tGMV as mediation variables, there is a higher mediation effect (PM = 6.77%).

Secondly, we consider the relationship between PRS and BAG, with Pack-year and tGMV as mediation variables. Figure 3f shown that both Pack-year and tGMV had a mediating effect (*β* = 0.0635, *p* = 0.028, PM = 36.039% for Pack-year; *β* = 0.0228, *p* = 0.32, PM = 77.01% for tGMV). When considering both BAG and tGMV as mediation variables, there is a higher mediation effect (PM = 84.72%).

### Functional annotations

**Figure 4.**
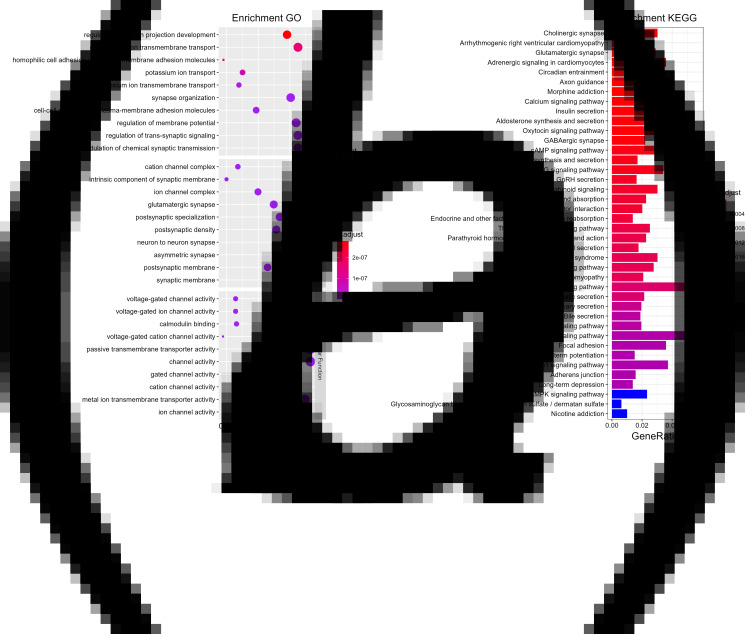
Functional annotations of smoking-related genes. **(a)** GO enrichment analysis results for PRS at PT_0.41. The top ten GO terms in cellular component (CC), molecular function (MF), and biological processes (BPs) were shown in different color dots. Count: the number of genes affected in PRS. p.adjust: *p*-value adjusted with FDR correction. **(b)** KEGG pathway enrichment analysis results for PRS at PT_0.41. Colored bars represented the top 50 KEGG pathway terms with corrected *p*-value. p.adjust: *p*-value adjusted with FDR correction.

To explore the potential molecular mechanisms underlying the BAG caused by smoking, we performed a functional enrichment analysis of genes included in the PRS calculation at *P_T_* = 0.04. The BPs were enriched in the modulation of chemical synaptic transmission, the regulation of neuron projection development, and the cell junction assembly. The CCs of PRS-calculated genes were significantly concentrated in cell-cell junction, cell leading edge and glutamatergic synapses. The ion channel activity, metal ion transmembrane transporter activity and GTPase regulator activity were significantly enriched MF. The top ten GO terms in BP, CC, and MF are shown below (Fig. 4a). The incorporated into PRS calculation genes significantly enriched in multiple KEGG pathways (Fig. 4b), and the top five pathways were cholinergic synapse, arrhythmogenic right ventricular cardiomyopathy, glutamatergic synapses, adrenergic signaling in cardiomyocytes, and circadian entrainment pathway, in addition, various neurotransmitter pathways, such as GABAergic synapse, and serotonergic synapse, were also enriched.

## Discussion

In this paper, we investigated the relationship between PRS, BAG, and GMV in smokers using data from the UK Biobank, and further annotated the gene related to smoking. Here, by conducting a BAG predictor, investigating the relationship between PRS, BAG, GMV and smoking parameters, and annotating the gene involved in our PRS, we verified our previous hypotheses. The BAG and PRS can measure brain ageing and genetic disparities between smokers and non-smokers, with smokers having greater brain ageing and genetic risk. We found that greater PRS was associated with bigger BAG, and this relationship was partly mediated by the amount of smoking and tGMV. A number of specific biological pathways are enriched in genes closely associated with smoking, particularly neurotransmitter and ion activity pathways, which may underlie the molecular mechanisms of smoking addiction and brain ageing, providing insight into the neurobiological underpinning, individualized neuroprotective treatment and intervention measures.

### The BAG is a valuable feature in smoking

Using the XGBoost combined with a nested five-fold CV, we achieved relatively high predictive accuracy in the test data (*r* = 0.900, MAE = 2.943). We found that the smoking group had a greater BAG than the non-smokers, and the gap increased with the severity of smoking, similar with PRS. These imply an association between accelerated brain ageing, more unhealthy smoking status, and higher genetic risk.

The rate of aging in an individual is determined by the interaction between various environmental, genetic, and epigenetic factors. The establishment of neuroanatomical features of the aging process is an emerging trend in neuroscience (Franke & Gaser, 2019). Brain age, used as a feature of brain ageing, could quantify the deviation of the individual brain from normal chronological ageing (Beck et al., 2022), which is well performed at the individual level and suited for clinical applications with broad sensitivity to psychiatric and neurological disorders (Ballester et al., 2022; Jongsiriyanyong & Limpawattana, 2018; Lee et al., 2021; Ramduny, Bastiani, Huedepohl, Sotiropoulos, & Chechlacz, 2022). Thus, in addition to simple univariate measures derived from the same imaging data, predicted brain age can provide a comprehensive view of healthy and pathological brain ageing (Millar et al., 2022). In the mediation analysis, we found that tGMV partially mediated the positive association between PRS and smoking with a mediation proportion of 5.59%, which was similar to that of BAG (5.04%), and the positive association between PRS and smoking with a mediation proportion of 65.38%. These indications suggest that BAG is a valuable reflection of overall brain atrophy, or that BAG is a valuable feature from the side.

### Smoking-related PRS

Smoking-related phenotypes, particularly nicotine dependence, are highly heritable, but the specific genetic variants (SNPs) associated with these phenotypes are controversial (Verde et al., 2011), and it has been shown in twin and family studies that smoking is not determined by a specific gene (Davies & Soundy, 2009). The PRS has been used as a global measure of risk score including any number of SNPs (Leonenko et al., 2021), which is a powerful tool for assessing genetic potency in some studies and significantly better than the GWAS (Lai et al., 2022; Vassos et al., 2017). Consequently, the method of assessing the relationship between genetic variation and smoking phenotype by PRS in this study is reasonable and has a simple calculation that is easy to understand.

In our studies, higher PRS was associated with more severe smoking status, similar to BAG. We found that PRS was positively correlated with BAG and negatively correlated with duration of quitting, implying that a higher risk at the genetic level may imply a more severe smoking habit. In mediation analysis, higher PRS was associated with more Pack-year, mediated by BAG, and associated with bigger BAG, mediated by Pack-year. These findings appear to indicate a vicious cycle in which increased smoking interacts with accelerated brain ageing, and such a cycle appears to be regulated by the genes associated with smoking.

Furthermore, several brain regions, significantly correlated with PRS, such as the superior frontal gyrus, middle frontal gyrus, anterior cingulate cortex, and nucleus accumbens, were involved in reward circuits (Haber & Knutson, 2010), which is a major component of the rewarding process and the neural basis for facilitative behavior, known as the mesocortical dopamine system (Gardner, 2011). Low dopamine is often the result of smoking addiction and is also a cause of Alzheimer's disease (Shan, 2011). These findings provide evidence that smoking addiction and smoking accelerates brain ageing or brain shrinkage.

It's important to note that the selection criteria used in pruning and thresholding should be carefully considered and justified to ensure the validity of the results. Additionally, as with any analysis, it's crucial to evaluate the limitations and potential sources of bias in the study design, including the choice of pruning and thresholding methods. Proper application and consideration of these methods can still yield valuable findings in understanding the genetic underpinnings of complex traits or diseases.

### Enrichment analysis of genes associated with smoking

In our study, smoking-related genes were enriched for glutaminergic synapses. Glutamate is the primary excitatory neurotransmitter in the brain (McGehee, Heath, Gelber, Devay, & Role, 1995). Neurochemical studies have demonstrated that nicotine, at concentrations achieved during smoking, can act at presynaptic receptors to enhance the release and function of glutamate (Monaghan, Bridges, & Cotman, 1989; Novak et al., 2010). And alterations in glutamatergic neurotransmission are thought to be involved in several neuropsychiatric disorders, such as SCZ, bipolar disorder, and alcoholism, as well as depression (Cheng et al., 2021; Novak et al., 2010; Vengeliene, Bilbao, Molander, & Spanagel, 2008; Verma & Shakya, 2022), which is consistent with the long-term depression pathway in our KEGG analysis. Structural and functional alterations in glutamatergic synapses may be associated with altered synaptic signaling and plasticity, commonly involved in developmental, psychiatric, and neurological disorders, including nicotine addiction (Van Spronsen & Hoogenraad, 2010). The main cytoskeletal component of dendritic spines is actin. A study has demonstrated that exposure to substances such as nicotine or morphine leads to sustained structural changes in the dendrites and dendritic spines on cells in brain regions involved in motivation and reward (e.g. the nucleus accumbens), judgment and inhibitory control of behavior (e.g. the prefrontal cortex) (Kalivas, 2009). Such structural changes are relevant to the actin-related pathways in our enrichment analysis results.

In our enrichment analysis, cholinergic synapses, GABAergic synapses, and serotonergic synaptic pathways were similarly significant and shown to be associated with addiction plasticity in previous studies (Shah & Aizenman, 2014). Also, in GWAS, we obtained suggestive-significant variants rs12650174 on the gene GRID2, the major excitatory neurotransmitter receptor in the mammalian brain.

In addition, multiple pathways were associated with ion channels in our enrichment analysis, such as ion channel activity, gated channel activity, and calcium signaling pathways, which are essential for neuronal function, triggering nerve impulses and neurotransmitter release, and linked to a variety of human neuropsychiatric disorders.

Voltage-gated potassium and sodium channels in the gated channel activity pathway are widely present in the central nervous system (CNS) and are associated with neuronal excitability (Zhang et al., 2021). Abnormally high or low neuronal excitability can lead to a variety of neurodegenerative and neuropsychiatric disorders, such as major depressive disorder (MDD), attention deficit hyperactivity disorder (ADHD), SCZ, AD, and autism spectrum disorder (ASD) (Wang, Ou, & Wang, 2017). In addition, calcium signaling pathways, increasing the risk of five major psychiatric disorders: ASD, ADHD, SCZ, and MDD (Gandal et al., 2018), play a key role in neurodegenerative diseases and neuropsychiatric disorders. For example, in AD, A*β* aggregation increases neuronal cytoplasmic calcium ions concentrations and further triggers synaptic dysfunction and neurodegeneration (Cross-Disorder Group of the Psychiatric Genomics Consortium*, 2019).

### Genome-wide association analysis

In genome-wide association analysis (Supplement), two SNPs showed genome-wide significant (*p* < 8.112419e-08) and six SNPs showed suggestive significant (*p* < 1.622484e-06)(online Supplementary Figure S6, Table S10). The sequence variant with the strongest association, rs199533 (*p* = 8.825e-19), tags the inverted form of the 17q21.31 inversion polymorphism, and the genes in which this SNP is located, *NSF, LRRC37A2*, are associated with progressive myoclonic epilepsies (de Jong et al., 2012; Koolen et al., 2006). Another genome-wide significant sequence variant, rs7542 (*p* = 1.689e-11), is located in *MAPK3*, a protein-coding gene associated with cholangiocarcinoma and pancreatic cancer (Cheng et al., 2021) where smoking play a definite role in, associated with developmental delays and intellectual disabilities. This may account for its association with BAG.

## Limitations

Some limitations should be considered. First, only structural MRI data was used in XGBoost, incorporating other modalities would lead to higher accuracy. And then it is worth noting that this study was conducted in an older group of participants and did not include younger subjects, which may lead to failure in predicting the brain age and polygenic risk of smokers with a wider age range. Second, PRS is calculated from SNPs and does not consider other genetic risks, such as CNVs or rare mutations. Finally, the results of this study were impossible to disentangle the causality of these associations among genes, brain ageing and smoking, and that could also be investigated in a future study.

## Conclusion

In summary, simplifying complex multivariate structural information from the entire brain and genome into a single indicator (i.e. BAG & PRS) enables better assessment of individual risks and is helpful to developing individualized neuroprotective treatment and intervention measures, which strengthen the understanding of the links between brain aging processes and potential molecular mechanism underlying smoking.

## Supporting information

Yang et al. supplementary material Yang et al. supplementary material

## Supplementary material

The supplementary material for this article can be found at https://doi.org/10.1017/S0033291723001812.
