# Supplementary material for: Associations between polygenic risk scores and accelerated brain ageing in smokers
Source: Psychol Med. 2023 Aug 9;53(16):7785–94. doi: 10.1017/S0033291723001812 (PMC10755245; doi:10.1017/S0033291723001812)
Supplement: Yang et al. supplementary material [file S0033291723001812sup001.docx]

**Associations between polygenic risk scores and accelerated brain ageing in smokers**

Zeyu Yang, Wei Zhao, Zeqiang Linli, Shuixia Guo, Jianfeng Feng

***Supplementary Materials***

**Contents**

[**Method**](#_Toc136284699)

[Smoker grouping 1](#_Toc136284700)

[Smoking parameters 1](#_Toc136284701)

[Brain Age Gap prediction 2](#_Toc136284702)

[Polygenic risk score 5](#_Toc136284703)

[Mediation analysis 6](#_Toc136284704)

[Other significant pathways in enrichment analysis 7](#_Toc136284705)

[Genome wide association study 9](#_Toc136284709)

[Sample relatedness in UK-biobank dataset 12](#_Toc136284710)

[Tables 14](#_Toc136284711)

[Figures 28](#_Toc136284721)

# Method

## Smoker grouping

The smokers were split into 6 subgroups based on their smoking characteristics (Smoking status and Smoking frequency, Table S1). Specifically, those who currently smoke on most or all days were classified as “Current smoker” (n = 1254); those who previously smoked on most or all days but occasionally still smoked were classified as “Ex-smoker1” (n = 240); those who previously smoked on most or all days and had stopped smoking at the time of the study were classified as “Ex-smoker2” (n = 6749); those who had smoked occasionally in the past and now and had smoked a total of at least 100 times were classified as “Light smoker1” (n = 418); those who used to smoke occasionally and smoked a total of at least 100 times but quit smoking currently were classified as “Light smoker2” (n = 3751); and those who had extremely lightly smoked and failed to meet the standards of light smokers (i.e., a total of at least 100 times in their lifetime) were classified as “Other smoker” (n = 6214).

## Smoking parameters

The other two key smoking parameters calculated were: i) pack-years was calculated as cigarettes per day divided by 20 and then times the number of years smoked and was only available for current and ex-smokers, measuring the amount of smoking; ii) quitting duration, which was calculated as age at the time of data collection minus the age when the participant stopped smoking on most days and this was only available for ex-smokers.

## Brain Age Gap prediction

In this study, extreme gradient boosting (XGBoost) predictors, a popular technique in recent brain age studies, was used to build a brain-age estimating model across all controls, with chronological age as the dependent variable and 166 regional GMV as independent variables, and was implemented using the R package xgboost (https://github. com/dmlc/xgboost). XGBoost is an implementation of gradientboosted trees designed for speed and performance; the final model is based on a collection of individual models. Compared to regular gradient boosting, which uses the loss function of the base model as a proxy for minimizing error, XGBoost computes second-order derivatives to provide information about the direction of gradients and how to obtain the minimum loss function. It also includes advanced regularization to reduce overfitting.

In this study, the tuning parameter ranges were set to maximum depth M = [2, 3, 4, 5, 6, 7, 8, 9, 10], number of estimators N = [50, 100, 150, 200, 250], and learning rate η = [0.01, 0.05, 0.1, 0.2, 0.5], and the default values were used for all other parameters. For each set of (M, N, η), the mean squared error was measured for each inner five-fold CV loop, and a mean value for all the five-fold inner loops was then obtained to indicate the inner prediction performance. The parameter set with the highest inner prediction accuracy among the inner five-fold CVs was chosen as the optimal parameter. Finally, all samples of the inner five-fold CV were trained with the best parameters, and the testing subjects of the outer five-fold CV were predicted. The training and testing procedures of the outer five-fold CV were repeated five times, with each subset used once as the testing set.

Therefore, a full nested five-fold CV loop finally produced five XGBoost predictors with optimal parameters, and all training data (controls) had a predicted age (interpreted as BrainAge). The BrainAge Gap can be computed by subtracting the chronological age from the estimated brain age (i.e., BrainAge – True age). The BrainAge of smokers was predicted using five XGBoost predictors and then averaged across the five predicted values as the final BrainAge of smokers, and the BrainAge Gap was also calculated for every smoker.

Specifically, we divided the brain into 166 regions based on the AAL3 brain atlas and extracted their grey matter volume (GMV), and regressed potential images for confounding variables such as gender, ethnicity, handedness, BMI, alcohol consumption, and TIV separately for regional GMV of all 166 regions using linear regression models. Then, an XGBoost prediction model was built to predict age in a non-smoking group of 𝑛 = 14,667 non-smoking subjects using the residuals of 166 linear regression models as features. To simultaneously train the predictive ability of the model and assess the generalization ability of the model, we used a nested 5-fold Cross Validation (5-fold CV) method to train the model, where the inner 5-fold CV was used to determine the optimal parameters and the outer 5-fold CV to estimate the generalization capability. The nested 5-fold CV loops produced five XGBoost prediction models with optimal parameters, and all training data had a predicted age (i.e. brain age). In the smoking group (N=18626), the brain age predicted using the 5 XGBoost prediction models and the mean of the 5 predicted values will be used as the final predicted brain age (BrainAge). Pearson's correlation coefficient (r), mean absolute error (MAE) and root mean squared error (RMSE) between predicted and chronological ages were calculated for the non-smoker and smoker groups respectively to assess the accuracy of the predictors.

Finally, BrainAge Gap (BAG) is defined as the difference between predicted age and chronological age (brain age - true age). Probably due to problems such as regression dilution and the non-Gaussian distribution of age, practical applications of brain age estimation often show age bias: BrainAge is overestimated in younger subjects and underestimated in older subjects, while BrainAge predictions are more accurate for subjects whose age is closer to the average age (of the training data set). Therefore, in order to avoid spurious correlations between BrainAge Gap and age-related variables in subsequent statistical analyses, a bias correction step is necessary. A common approach is to apply a linear regression model to remove the effect of chronological age on the estimated BrainAge. In the present study, we used the following BrainAge correction model in the non-smoker group.

$$BrainAge=\beta_{0}+\beta_{1}age+\beta_{2}age^{2}+\varepsilon$$

The residuals of this regression equation represent the difference between BrainAge and chronological age after controlling for confounders, including linear and quadratic effects of chronological age, which we refer to as the Corrected BrainAge Gap and are calculated using the following formula (2). Further, Corrected BrainAge can be calculated using the following formula(3).

$$Corrected\text{ }BrainAge\text{ }Gap=BrainAge-(\beta_{0}+\beta_{1}age+\beta_{2}age^{2})$$

$$Corrected\text{ }BrainAge=Chronological\text{ }Age+Corrected\text{ }BrainAge\text{ }Gap$$

The BrainAge Gap (BAG) used in the statistical analysis was adjusted for bias unless stated otherwise.

## Polygenic risk score

In our calculation of PRS, the base data was a meta-analysis of over 30 genome wide association studies (GWAS) in over 1.2 million participants with European ancestry on nicotine and substance use (https://doi.org/10.1038/s41588-018-0307-5). Specifically, it targeted different stages and kinds of substance use from initiation (smoking initiation and age of regular smoking initiation) to regular use (drinks per week and cigarettes per day) to cessation (smoking cessation). The GWAS included have all been imputed to Haplotype Reference Consortium, 1000 Genomes or a combination including more specific reference panels. (more see <https://conservancy.umn.edu/handle/11299/201564>)

The PRS was generated using PRSice-2 software (choishingwan.github.io/PRSice/) and calculated in the target sample according to the following equation:

$$PRS_{P_{T},j}=\sum_{i=1}^{m} \beta_{i}G_{ij}$$

where, 𝑃_𝑇_ represents the p-value threshold, 𝑖=1,2, ..., 𝑚 denotes the 𝑖-th SNP under the 𝑃_𝑇_ threshold, 𝛽_𝑖_ denotes the effect size of the 𝑖-th SNP, 𝑗=1,2, ..., 𝑛 represents the 𝑗-th sample, 𝐺_𝑖𝑗_ =0,1,2 denotes the number of risk alleles for the 𝑖-th SNP in the 𝑗-th sample. The PRS at 𝑃_𝑇_ refers to the cumulative polygenic risk, that is, the weighted sum of the SNPs with GWAS p value less than 𝑃_𝑇_.

## Mediation analysis

To verify whether there is a mediating effect among Pack-year, BAG and PRS, a standard three-variable path mediation model was implemented using the R package “mediation”, and the specific model as follows:

(1) 𝑌 = 𝑘_1_ + 𝜏𝑋 + ω_1_*Covar* + 𝜀_1_ .

(2) 𝑍 = 𝑘_2_ + 𝛼𝑋 + ω_2_*Covar* +𝜀_2_ .

(3) 𝑌 = 𝑘_4_ + 𝜏′𝑋 + 𝛽’𝑍 + ω_4_*Covar* + 𝜀_4_ .

where *X* is the predictor variable, *Y* is the response variable, and *Z* is the mediating variable, *Covar* is a covariate, as our hypotheses;

Further, we used a 4-variable mediation analysis model to test whether the relationship between PRS and Pack-year was mediated by tGMV and BAG, and whether the relationship between PRS and BAG was mediated by Pack-year and tGMV. The specific model was as follow:

(1) 𝑌 = 𝑘_1_ + 𝜏𝑋 + ω_1_*Covar* + 𝜀_1_ .

(2) 𝑍 = 𝑘_2_ + 𝛼𝑋 + ω_2_*Covar* + 𝜀_2_ .

(3) 𝑌 = 𝑘_3_ + δ𝑍 + ω_3_*Covar* + 𝜀_3_ .

(4) 𝑀= 𝑘_4_ + 𝜁𝑋 + λ𝑍 + ω_4_*Covar* + 𝜀_4_ .

(5) 𝑌 = 𝑘_5_ + 𝜏′𝑋 + 𝛾𝑀 + 𝜂𝑍 + ω_5_*Covar* + 𝜀_5_ .

where *X* is the predictor variable, *Y* is the response variable, and *M* and *Z* are the mediating variables, *Covar* is a covariate;

The significance of the mediation, that is, whether the relationship had been significantly reduced with the inclusion of the mediator, was estimated using the bias-corrected bootstrap approach (with 1000 random samplings). Confounding variables, as in the association analysis, were regressed in the mediation model. The percentage of the mediation effect (PM) that could be explained by the mediator (indirect effect) was measured using the formula: 100% × (𝜏−𝜏′)/(𝜏).

Since not all participants had Pack-year and PRS data, the mediation analysis was restricted to smokers who had Pack-year data (N=5799). It is worth mentioning that significant mediation effects indicate to what extent is the correlation between the independent and dependent variables related to the mediating variable, and that this is strictly a measure of association and does not imply causal relationships.

## Other significant pathways in enrichment analysis

### Morphine addiction

Morphine addiction can cause alterations in dopaminergic neurotransmission, and stress depression is associated with reduced dopaminergic nerve function, suggesting that morphine addiction is closely related to stress depression affective psychosis and that they may share one or more of the same neurobiological mechanisms. The region, from the ventral tegmental area (VTA) to the nucleus accumbens (NAc), the medial prefrontal cortex (mPFC), the amygdala (Amy), the hippocampus (Hip) and the striatum (ST), play an important role in morphine addiction and stress depression.

### ErbB signaling pathway

The ErbB receptor tyrosine kinase family consists of four cell membrane receptors that are activated following ligand binding and receptor dimerization. Insufficient ErbB signaling is associated with the development of neurodegenerative diseases, such as multiple sclerosis and Alzheimer's disease. Additionally, several types of cancer are associated with the mutation or increased expression of members of the ErbB family including lung, breast, stomach, and pancreatic cancers. Because ErbB family members are critical factors in the development and malignancy of these tumors, they have been important therapeutic targets.

### Focal adhesion kinase

Focal adhesion kinase (FAK) is a non-receptor protein tyrosine kinase that is overexpressed and activated in several cancers, including SCLC, and contributing to cancer progression and metastasis through its important role in cell proliferation, survival, adhesion, spreading, migration, and invasion. Increased FAK expression or activity has been observed by various methods in many human cancers, including lung cancer.

Someone believes that Glutamate is the real workhorse of all transmitters in the brain. Dopamine is the more popularly known neurotransmitter, a lack of which contributes to depression, anxiety, attention deficit hyperactivity disorder and Parkinson's disease—but it actually accounts for less than 5 percent of all synaptic activity. By contrast, glutamate accounts for about 50 percent of this activity and is especially involved in the reward-motivation circuits integral to addiction.(from [Study finds neurotransmitter may play a role in alcohol relapse, addiction (medicalxpress.com)](https://medicalxpress.com/news/2018-02-neurotransmitter-role-alcohol-relapse-addiction.html) by Indiana University)

## Genome wide association study

Genome wide association study (GWAS) is a genotype-phenotype association test for a dense set of hundreds of thousands to millions of genetic variants across the genome Genotype-phenotype association tests are performed on hundreds of thousands to millions of genetic variants across the genome to investigate common genetic variants in disease or to identify heritable quantitative traits for disease risk factors. GWAS has many advantages . First, the genetic loci identified by GWAS genetic loci identified by GWAS often involve new disease-causing genes of unknown function or previously unanticipated, and subsequent studies of these genes may reveal new biological aspects of the disease. Subsequent studies of these genes may reveal new biological mechanisms of disease, which in turn may provide insight into the structure of disease susceptibility. Secondly. GWAS can help to discover new drug targets and disease biomarkers. Finally, the genetic variants identified by GWAS can be used to identify high GWAS can be used to identify individuals at high risk of certain diseases for early detection, prevention, or treatment, thus contributing to the development of personalized medicine. The genetic variants identified by GWAS can be used to identify individuals at high risk of certain diseases for early detection, prevention, or treatment, thus contributing to personalized medicine.

In order to look for such sequence variants, we ran a genome wide association scan (GWAS) in the UK Biobank sample on Pack-year which quantifies smoking and associates with accelerated brain ageing using Plink software.

As for GWAS quality control, SNPs with minor allele frequencies (MAF) <1%, call rates <95%, Hardy-Weinberg equilibrium p<10^-5^ were excluded from analysis, and individuals with excessive missingness >5% and sex mismatches >0.90 were excluded from the study.

To protect against potential confounding effects, we adjusted for potential nuisance variables, such as sex, age, TIV, handedness, BMI, alcohol consumption and the first ten components generated. In our GWAS, the exome-wide significance threshold was set to be 0.05/number of the tests, which is 0.05/616,339 = 8.112419e-08 and the suggestive significance was set to be 1/number of the tests, which is 1/616,339 = 1.622484e-06, as suggested by a previous GWAS study.

Finally, two genome-wide significance associations and six suggestive significance association were identified (Table S9, Figure S3, S4). In our GWAS, Genomic Inflation Factor = 1.082, indicating that there is no population stratification, and it is harder to have a false positive result, and there is no need to correct the population stratification.

The sequence variant with the strongest association, rs199533 (p=8.825e-19), is located at 17q21.31, a region involved in inversion polymorphism. This inversion spans ~1 Mb and includes 10 genes, including *CRHR1, MAPT, NSF* and a protein-coding gene (*LRRC37A2* leucine-rich repeat sequence). Among them, the *MAPT* gene is associated with several dementias, the *CRHR1* gene is closely related to the development of depression, anxiety, suicide, bipolar disorder and other psychiatric disorders of affective disorders, and the gene *NSF*, in which the sequence variant rs199533 is located, *LRRC37A2* is associated with progressive myoclonic epilepsies. Due to extensive linkage disequilibrium (LD) in the 17q21.31 inversion region, markers of various associations in this region often differ in studies.

Another genome-wide significant sequence variant, rs7542 (p=1.689e-11), is located in ***MAPK3***, a protein-coding gene, and the diseases associated with ***MAPK3*,** including pancreatic and cholangiocarcinoma cancer. Individuals with 16p11.2, the chromosomal location where the sequence variant rs7542 is located, deletion syndrome typically have developmental delays and intellectual disabilities, and most also have at least some features of autism spectrum disorders, which may account for its association with positive BAG. Smoking is currently the only recognized risk factor with a definite role in the development of pancreatic cancer. Numerous prospective and case-control studies have shown that the risk ratio of death in pancreatic cancer patients who smoke versus non-smokers is between 1.6 and 3.1:1, and that the amount of smoking is positively associated with the development of pancreatic cancer.

The six suggestive significant sequence variants were rs864736 (p=9.715e-08), rs12650174 (p=9.475e-07), rs75298305 (p=8.422e-07), rs12146713 (p=5.368e-07), rs11618612 (p=7.590e-07), and rs6119728 (p=1.081e-07);

rs864736 is located near *KCNK2* (also known as *TREK1*), the eQTL for the *KCNK2* gene. This gene regulates the entry of immune cells into the central nervous system (CNS) and controls CNS inflammation, which is associated with cortical atrophy and cognitive decline. Significantly associated with posterior margin of the left corpus callosum, intraparietal and central sulcus sulci and grey matter thickness.

The gene in which rs12650174 is located is *GRID2*, which encodes a protein that is a member of the family of ionotropic glutamate receptors, the major excitatory neurotransmitter receptors in the mammalian brain.

The gene nearest to rs12146713 on chromosome 12 (associated with medial nuclei volume) is *NUAK1*, which regulates the Tau protein level. Cerebral Tau accumulation is a defining characteristic of Alzheimer’s disease (AD) and other neurodegenerative disorders.

## Sample relatedness in UK-biobank dataset

Given the nested nature of the data and the fact that many related samples were included in the UKB, we adjusted BAG prediction and association analysis for sample relatedness (the first 5 principal from components genetic ancestry analysis) which could measure the independence of the family members. Specifically, the gray matter of images were segmented after the common preprocessing procedure and partitioned into 166 regions of interest based on the automated anatomical labeling 3 (AAL3) atlas, which further were residualized for sex, ethnicity, handedness, BMI, scanning site, alcohol consumption, TIV and sample relatedness using linear regression models and then input to XGBoost predictor. And in the association analysis, sample relatedness was added to the covariates to correct for confound effect. All this adjustment only had a small effect on our results. In the Brain Age Gap prediction(Table S10, Figure S13-S15), the brain age predicted by considering sample relatedness was highly correlated with age without considering sample relatedness (r= 0.9636619, p < 2.2e-16 in test data, Figure S15).

# Tables

## Table S1. Demographic variables

|  | **Non-smoker**  **(n = 14667)** | **Smoker**  **(n = 18626)** | **p-value** |
| --- | --- | --- | --- |
| **Age (mean (SD))** | 63.12 (7.52) | 64.20 (7.51) | <0.001 |
| **Male (%)** | 6418 (43.8) | 9233 (49.6) | <0.001 |
| **Handedness: right (%)** | 13087 (89.2) | 16535 (88.8) | 0.195 |
| **BMI (mean (SD))** | 26.33 (4.19) | 26.62 (4.18) | <0.001 |
| **Alcohol consumption (mean (SD))** | 27.06 (29.74) | 40.03 (38.59) | <0.001 |
| **Pack year (mean (SD))** | / | 18.62 (15.06) | / |
| **TIV (mean (SD))**  **Education (mean (SD))** | 1549.29 (152.67) | 1564.88 (151.82) | <0.001 |
|  | 4.40 (1.89) | 4.35 (1.94) | 0.027 |

Note: TIV was calculated as the sum of grey matter, white matter, and cerebrospinal fluid volumes in natural space. Education was the top qualification participants ,encoded using Data-Coding 100305 (https://biobank.ndph.ox.ac.uk/showcase/coding.cgi?id=100305).

## Table S2. Demographic variables of subgroups of smokers

|  | **Current Smoker**  **(n = 1254)** | **Ex-smoker1**  **(n = 240)** | **Ex-smoker2**  **(n = 6749)** | **Light Smoker1**  **(n = 418)** | **Light Smoker2**  **(n = 3751)** | **Other Smoker**  **(n = 6214)** |
| --- | --- | --- | --- | --- | --- | --- |
| **Age (mean (SD))** | 61.84 (7.29) | 62.59 (7.37) | 65.64 (7.21) | 61.72 (7.64) | 64.36 (7.48) | 63.25 (7.58) |
| **Male (%)** | 644 (51.4) | 130 (54.2) | 3557 (52.7) | 259 (62.0) | 1918 (51.1) | 2725 (43.9) |
| **Handedness: right (%)** | 1108 (88.4) | 212 (88.3) | 5966 (88.4) | 379 (90.7) | 3311 (88.3) | 5559 (89.5) |
| **BMI (mean (SD))** | 26.34 (4.17) | 26.76 (4.64) | 27.37 (4.24) | 26.57 (3.52) | 26.33 (3.90) | 26.04 (4.19) |
| **Alcohol consumption (mean (SD))** | 47.89 (51.16) | 47.67 (43.01) | 45.83 (42.27) | 51.44 (37.86) | 39.36 (34.96) | 31.50 (30.96) |
| **Pack year (mean (SD))** | 24.08 (15.91) | 17.38 (13.93) | 17.65 (14.72) | / | / | / |
| **TIV (mean (SD))** | 1561.15 (154.18) | 1583.90 (157.65) | 1570.75 (151.42) | 1605.19 (152.20) | 1566.21 (149.63) | 1555.01 (152.04) |
| **Education (mean (SD))** | 4.16 (2.01) | 4.38 (1.87) | 4.27 (2.00) | 4.24 (1.91) | 4.30 (1.97) | 4.52 (1.84) |
| **Quitting duration (mean (SD))** | / | 19.09 (9.66) | 29.28 (11.50) | / | / | / |

## Table S3. Characteristics of smoking subgroups

| Groups | N | Smoking status | | Smoking frequency | | Note |
| --- | --- | --- | --- | --- | --- | --- |
|  |  | **Current** | **Past** | **Regular** | **Occasional** |  |
| Current smoker | 1254 | √ |  | √ |  |  |
| Ex-smoker1 | 240 |  | √ | √ |  | Current smoking occasionally |
| Ex-smoker2 | 6749 |  | √ | √ |  |  |
| Light smoker1 | 418 | √ |  |  | √ | At least 100 smokes in lifetime |
| Light smoker2 | 3751 | √ | √ |  | √ | At least 100 smokes in lifetime |
| Oher smoker | 6214 | √ | √ |  | √ | Less than 100 smokes in lifetime |
| Non-smoker | 14667 |  |  |  |  | Never smoking |

## Table S4. The anatomical regions defined in each hemisphere and their label in the automated anatomical labeling atlas AAL3.

| No. | Anatomical description | Label | Possible | Network |
| --- | --- | --- | --- | --- |
|  |  | **aal2.nii.gz** | **abbreviation** |  |
| 1,2 | Precentral gyrus | Precentral | PreCG | Sensorimotor |
| 3, 4 | Superior frontal gyrus, dorsolateral | Frontal_Sup | SFG | Frontal |
| 5, 6 | Middle frontal gyrus | Frontal_Mid | MFG | Frontal |
| 7, 8 | Inferior frontal gyrus, opercular part | Frontal_Inf_Oper | IFGoperc | Frontal |
| 9, 10 | Inferior frontal gyrus, triangular part | Frontal_Inf_Tri | IFGtriang | Frontal |
| 11, 12 | IFG pars orbitalis, | Frontal_Inf_Orb | IFGorb | Frontal |
| 13, 14 | Rolandic operculum | Rolandic_Oper | ROL | Frontal |
| 15, 16 | Supplementary motor area | Supp_Motor_Area | SMA | Sensorimotor |
| 17, 18 | Olfactory cortex | Olfactory | OLF | Frontal |
| 19, 20 | Superior frontal gyrus, medial | Frontal_Sup_Med | SFGmedial | Frontal |
| 21, 22 | Superior frontal gyrus, medial orbital | Frontal_Med_Orb | PFCventmed | Frontal |
| 23, 24 | Gyrus rectus | Rectus | REC | Frontal |
| 25, 26 | Medial orbital gyrus | OFCmed | OFCmed | Frontal |
| 27, 28 | Anterior orbital gyrus | OFCant | OFCant | Frontal |
| 29, 30 | Posterior orbital gyrus | OFCpost | OFCpost | Frontal |
| 31, 32 | Lateral orbital gyrus | OFClat | OFClat | Frontal |
| 33, 34 | Insula | Insula | INS | Subcortical |
| 35, 36 | Anterior cingulate & paracingulate gyri | Cingulate_Ant | ACC | Frontal |
| 37, 38 | Middle cingulate & paracingulate gyri | Cingulate_Mid | MCC | Frontal |
| 39, 40 | Posterior cingulate gyrus | Cingulate_Post | PCC | Parietal |
| 41, 42 | Hippocampus | Hippocampus | HIP | Temporal |
| 43, 44 | Parahippocampal gyrus | ParaHippocampal | PHG | Temporal |
| 45, 46 | Amygdala | Amygdala | AMYG | Subcortical |
| 47, 48 | Calcarine fissure and surrounding cortex | Calcarine | CAL | Occipital |
| 49, 50 | Cuneus | Cuneus | CUN | Occipital |
| 51, 52 | Lingual gyrus | Lingual | LING | Occipital |
| 53, 54 | Superior occipital gyrus | Occipital_Sup | SOG | Occipital |
| 55, 56 | Middle occipital gyrus | Occipital_Mid | MOG | Occipital |
| 57, 58 | Inferior occipital gyrus | Occipital_Inf | IOG | Occipital |
| 59, 60 | Fusiform gyrus | Fusiform | FFG | Temporal |
| 61, 62 | Postcentral gyrus | Postcentral | PoCG | Sensorimotor |
| 63, 64 | Superior parietal gyrus | Parietal_Sup | SPG | Parietal |
| 65, 66 | Inferior parietal gyrus, excluding supramarginal and angular gyri | Parietal_Inf | IPG | Parietal |
| 67, 68 | SupraMarginal gyrus | SupraMarginal | SMG | Parietal |
| 69, 70 | Angular gyrus | Angular | ANG | Parietal |
| 71, 72 | Precuneus | Precuneus | PCUN | Parietal |
| 73, 74 | Paracentral lobule | Paracentral_Lobule | PCL | Parietal |
| 75, 76 | Caudate nucleus | Caudate | CAU | Subcortical |
| 77, 78 | Lenticular nucleus, Putamen | Putamen | PUT | Subcortical |
| 79, 80 | Lenticular nucleus, Pallidum | Pallidum | PAL | Subcortical |
| 81, 82 | Thalamus | Thalamus | THA | Subcortical |
| 83, 84 | Heschl’s gyrus | Heschl | HES | Temporal |
| 85, 86 | Superior temporal gyrus | Temporal_Sup | STG | Temporal |
| 87, 88 | Temporal pole: superior temporal gyrus | Temporal_Pole_Sup | TPOsup | Temporal |
| 89, 90 | Middle temporal gyrus | Temporal_Mid | MTG | Temporal |
| 91, 92 | Temporal pole: middle temporal gyrus | Temporal_Pole_Mid | TPOmid | Temporal |
| 93, 94 | Inferior temporal gyrus | Temporal_Inf | ITG | Temporal |
| 95, 96 | Crus I of cerebellar hemisphere | Cerebellum_Crus1 | CERCRU1 | Cerebellum |
| 97, 98 | Crus II of cerebellar hemisphere | Cerebellum_Crus2 | CERCRU2 | Cerebellum |
| 99, 100 | Lobule III of cerebellar hemisphere | Cerebellum_3 | CER3 | Cerebellum |
| 101, 102 | Lobule IV, V of cerebellar hemisphere | Cerebellum_4_5 | CER4_5 | Cerebellum |
| 103, 104 | Lobule VI of cerebellar hemisphere | Cerebellum_6 | CER6 | Cerebellum |
| 105, 106 | Lobule VIIB of cerebellar hemisphere | Cerebellum_7b | CER7b | Cerebellum |
| 107, 108 | Lobule VIII of cerebellar hemisphere | Cerebellum_8 | CER8 | Cerebellum |
| 109, 110 | Lobule IX of cerebellar hemisphere | Cerebellum_9 | CER9 | Cerebellum |
| 111, 112 | Lobule X of cerebellar hemisphere | Cerebellum_10 | CER10 | Cerebellum |
| 113 | Lobule I, II of vermis | Vermis_1_2 | VER1_2 | Cerebellum |
| 114 | Lobule III of vermis | Vermis_3 | VER3 | Cerebellum |
| 115 | Lobule IV, V of vermis | Vermis_4_5 | VER4_5 | Cerebellum |
| 116 | Lobule VI of vermis | Vermis_6 | VER6 | Cerebellum |
| 117 | Lobule VII of vermis | Vermis_7 | VER7 | Cerebellum |
| 118 | Lobule VIII of vermis | Vermis_8 | VER8 | Cerebellum |
| 119 | Lobule IX of vermis | Vermis_9 | VER9 | Cerebellum |
| 120 | Lobule X of vermis | Vermis_10 | VER10 | Cerebellum |
| 121, 122 | Thalamus, Anteroventral Nucleus | Thal_AV | tAV | Subcortical |
| 123, 124 | Lateral posterior | Thal_LP | tLP | Subcortical |
| 125, 126 | Ventral anterior | Thal_VA | tVA | Subcortical |
| 127, 128 | Ventral lateral | Thal_VL | tVL | Subcortical |
| 129, 130 | Ventral posterolateral | Thal_VPL | tVPL | Subcortical |
| 131, 132 | Intralaminar | Thal_IL | tIL | Subcortical |
| 133, 134 | Reuniens | Thal_Re | tRe | Subcortical |
| 135, 136 | Mediodorsal medial magnocellular | Thal_MDm | tMDm | Subcortical |
| 137, 138 | Mediodorsal lateral parvocellular | Thal_MDl | tMDl | Subcortical |
| 139, 140 | Lateral geniculate | Thal_LGN | tLGN | Subcortical |
| 141, 142 | Medial Geniculate | Thal_MGN | tMGN | Subcortical |
| 143, 144 | Pulvinar anterior | Thal_PuA | tPuA | Subcortical |
| 145, 146 | Pulvinar medial | Thal_PuM | tPuM | Subcortical |
| 147, 148 | Pulvinar lateral | Thal_PuL | tPuL | Subcortical |
| 149, 150 | Pulvinar inferior | Thal_PuI | tPuI | Subcortical |
| 151, 152 | Anterior cingulate cortex, subgenual | ACC_sub | ACCsub | Frontal |
| 153, 154 | Anterior cingulate cortex, pregenual | ACC_pre | ACCpre | Frontal |
| 155, 156 | Anterior cingulate cortex, supracallosal | ACC_sup | ACCsup | Frontal |
| 157, 158 | Nucleus accumbens | N_Acc | Nacc | Brainstem |
| 159, 160 | Ventral tegmental area | VTA | VTA | Brainstem |
| 161, 162 | Substantia nigra, pars compacta | SN_pc | SNpc | Brainstem |
| 163, 164 | Substantia nigra, pars reticulata | SN_pr | SNpr | Brainstem |
| 165, 166 | Red nucleus | Red_N | RedN | Brainstem |
| 167, 168 | Locus coeruleus | LC | LC | Brainstem |
| 169 | Raphe nucleus, dorsal | Raphe_D | RapheD | Brainstem |
| 170 | Raphe nucleus, median | Raphe_M | RapheM | Brainstem |

Note. Column 4 provides a set of possible abbreviations for the anatomical descriptions. The original numbers in AAL2 for the anterior cingulate cortex (35, 36) and thalamus (81, 82) are left empty in AAL3, as those voxels were substituted by the new subdivisions (Thalamic nuclei: 121-151; anterior cingulate cortex: 151-156). Thus, the total number of parcellations in AAL3 is 166, with maximum label number 170. This ensures that most of the numbers used in AAL2 remain the same in AAL3, while AAL3 mainly adds new areas starting at number 121.

## Table S5. Results of correlation analysis between PRS and smoking at different P_T_ thresholds.

| **P_T_** | **r** | **p-value** | **P_T_** | **r** | **p-value** | **P_T_** | **r** | **p-value** | **P_T_** | **r** | **p-value** |
| --- | --- | --- | --- | --- | --- | --- | --- | --- | --- | --- | --- |
| 5e.08 | 0.00422 | 0.52702 | 0.125 | 0.02110 | 0.00157 | 0.250 | 0.01917 | 0.00407 | 0.375 | 0.01892 | 0.00459 |
| 0.005 | 0.01794 | 0.00717 | 0.130 | 0.02141 | 0.00134 | 0.255 | 0.01912 | 0.00418 | 0.380 | 0.01885 | 0.00474 |
| 0.010 | 0.01709 | 0.01043 | 0.135 | 0.02050 | 0.00213 | 0.260 | 0.01922 | 0.00398 | 0.385 | 0.01859 | 0.00535 |
| 0.015 | 0.01827 | 0.00619 | 0.140 | 0.02006 | 0.00264 | 0.265 | 0.01896 | 0.00450 | 0.390 | 0.01850 | 0.00557 |
| 0.020 | 0.01905 | 0.00430 | 0.145 | 0.02018 | 0.00249 | 0.270 | 0.01884 | 0.00476 | 0.395 | 0.01836 | 0.00593 |
| 0.025 | 0.02141 | 0.00134 | 0.150 | 0.01997 | 0.00276 | 0.275 | 0.01897 | 0.00448 | 0.400 | 0.01812 | 0.00664 |
| 0.030 | 0.01994 | 0.00281 | 0.155 | 0.02047 | 0.00216 | 0.280 | 0.01867 | 0.00515 | 0.405 | 0.01812 | 0.00661 |
| 0.035 | 0.02112 | 0.00155 | 0.160 | 0.02086 | 0.00178 | 0.285 | 0.01883 | 0.00478 | 0.410 | 0.01811 | 0.00665 |
| 0.040 | 0.02305 | 0.00055 | 0.165 | 0.02107 | 0.00159 | 0.290 | 0.01892 | 0.00457 | 0.415 | 0.01833 | 0.00601 |
| 0.045 | 0.02276 | 0.00065 | 0.170 | 0.02060 | 0.00202 | 0.295 | 0.01886 | 0.00471 | 0.420 | 0.01830 | 0.00610 |
| 0.050 | 0.02277 | 0.00064 | 0.175 | 0.02060 | 0.00202 | 0.300 | 0.01884 | 0.00475 | 0.425 | 0.01814 | 0.00656 |
| 0.055 | 0.02167 | 0.00117 | 0.180 | 0.02061 | 0.00201 | 0.305 | 0.01877 | 0.00492 | 0.430 | 0.01791 | 0.00727 |
| 0.060 | 0.02106 | 0.00160 | 0.185 | 0.02113 | 0.00154 | 0.310 | 0.01887 | 0.00468 | 0.435 | 0.01781 | 0.00761 |
| 0.065 | 0.02044 | 0.00219 | 0.190 | 0.02125 | 0.00145 | 0.315 | 0.01910 | 0.00422 | 0.440 | 0.01751 | 0.00871 |
| 0.070 | 0.01970 | 0.00315 | 0.195 | 0.02106 | 0.00160 | 0.320 | 0.01949 | 0.00349 | 0.445 | 0.01784 | 0.00750 |
| 0.075 | 0.02110 | 0.00157 | 0.200 | 0.02117 | 0.00152 | 0.325 | 0.01921 | 0.00400 | 0.450 | 0.01787 | 0.00741 |
| 0.080 | 0.02085 | 0.00179 | 0.205 | 0.02090 | 0.00173 | 0.330 | 0.01888 | 0.00468 | 0.455 | 0.01770 | 0.00798 |
| 0.085 | 0.02054 | 0.00208 | 0.210 | 0.02093 | 0.00171 | 0.335 | 0.01909 | 0.00422 | 0.460 | 0.01753 | 0.00862 |
| 0.090 | 0.02071 | 0.00191 | 0.215 | 0.02039 | 0.00224 | 0.340 | 0.01891 | 0.00460 | 0.465 | 0.01757 | 0.00846 |
| 0.095 | 0.02006 | 0.00265 | 0.220 | 0.02045 | 0.00218 | 0.345 | 0.01900 | 0.00442 | 0.470 | 0.01739 | 0.00916 |
| 0.100 | 0.02042 | 0.00222 | 0.225 | 0.02067 | 0.00195 | 0.350 | 0.01885 | 0.00472 | 0.475 | 0.01748 | 0.00883 |
| 0.105 | 0.01985 | 0.00294 | 0.230 | 0.01992 | 0.00284 | 0.355 | 0.01898 | 0.00444 | 0.480 | 0.01732 | 0.00947 |
| 0.110 | 0.02057 | 0.00205 | 0.235 | 0.01947 | 0.00353 | 0.360 | 0.01906 | 0.00428 | 0.485 | 0.01745 | 0.00892 |
| 0.115 | 0.02113 | 0.00154 | 0.240 | 0.01959 | 0.00333 | 0.365 | 0.01891 | 0.00461 | 0.490 | 0.01749 | 0.00875 |
| 0.120 | 0.02132 | 0.00140 | 0.245 | 0.01920 | 0.00400 | 0.370 | 0.01897 | 0.00448 | 0.495 | 0.01747 | 0.00885 |

## Table S6. Results of partial correlation analysis between PRS and Pack-year under TOP10 threshold

| **P_T_** | **r** | **p-value** | **n** |
| --- | --- | --- | --- |
| 0.040 | 0.02305 | 0.00055 | 16363 |
| 0.050 | 0.02277 | 0.00064 | 16363 |
| 0.045 | 0.02276 | 0.00065 | 16363 |
| 0.055 | 0.02167 | 0.00117 | 16363 |
| 0.130 | 0.02141 | 0.00134 | 16363 |
| 0.025 | 0.02141 | 0.00134 | 16363 |
| 0.120 | 0.02132 | 0.00140 | 16363 |
| 0.190 | 0.02125 | 0.00145 | 16363 |
| 0.200 | 0.02117 | 0.00152 | 16363 |
| 0.115 | 0.02113 | 0.00154 | 16363 |

## Table S7. Results of correlation analysis between PRS and GM of brain regions (P_T_=0.41).

| **NO.in AAL3** | **LABEL in AAL3** | **r** | **p** |
| --- | --- | --- | --- |
| 134 | Thal_Re_R | -0.02926 | 1.16E-05 |
| 122 | Thal_AV_R | -0.02873 | 1.67E-05 |
| 12 | Frontal_Inf_Orb_2_R | -0.028 | 2.72E-05 |
| 6 | Frontal_Mid_2_R | -0.02668 | 6.39E-05 |
| 32 | OFClat_R | -0.02604 | 9.54E-05 |
| 46 | Amygdala_R | -0.02583 | 0.000108 |
| 31 | OFClat_L | -0.02414 | 0.000298 |
| 22 | Frontal_Med_Orb_R | -0.02399 | 0.000325 |
| 86 | Temporal_Sup_R | -0.02397 | 0.000328 |
| 45 | Amygdala_L | -0.02328 | 0.000485 |
| 90 | Temporal_Mid_R | -0.02237 | 0.000803 |
| 24 | Rectus_R | -0.02215 | 0.0009 |
| 126 | Thal_VA_R | -0.02184 | 0.001066 |
| 66 | Parietal_Inf_R | -0.02124 | 0.001461 |
| 153 | ACC_pre_L | -0.02107 | 0.001592 |
| 11 | Frontal_Inf_Orb_2_L | -0.02087 | 0.001765 |
| 89 | Temporal_Mid_L | -0.02066 | 0.001963 |
| 5 | Frontal_Mid_2_L | -0.02026 | 0.002397 |
| 44 | ParaHippocampal_R | -0.02009 | 0.002606 |
| 18 | Olfactory_R | -0.01995 | 0.00279 |
| 154 | ACC_pre_R | -0.01994 | 0.002813 |
| 136 | Thal_MDm_R | -0.0199 | 0.002865 |
| 133 | Thal_Re_L | -0.01985 | 0.002939 |
| 10 | Frontal_Inf_Tri_R | -0.01979 | 0.003028 |
| 33 | Insula_L | -0.01972 | 0.003125 |
| 138 | Thal_MDl_R | -0.0194 | 0.003649 |
| 65 | Parietal_Inf_L | -0.01884 | 0.004755 |
| 9 | Frontal_Inf_Tri_L | -0.01884 | 0.004765 |
| 26 | OFCmed_R | -0.01878 | 0.004886 |
| 135 | Thal_MDm_L | -0.01874 | 0.004977 |
| 34 | Insula_R | -0.01819 | 0.006409 |
| 55 | Occipital_Mid_L | -0.01809 | 0.006721 |
| 139 | Thal_LGN_L | -0.01804 | 0.006867 |
| 94 | Temporal_Inf_R | -0.01802 | 0.006919 |
| 152 | ACC_sub_R | -0.0177 | 0.008008 |
| 29 | OFCpost_L | -0.01768 | 0.008054 |
| 21 | Frontal_Med_Orb_L | -0.01765 | 0.008165 |
| 141 | Thal_MGN_L | -0.01762 | 0.008294 |
| 128 | Thal_VL_R | -0.01751 | 0.008682 |

## Table S8. The difference in BrainAge Gap between the smoker subgroups and the non-smoker group

|  | Mean difference (CI) | t-value | p-value | Cohen’s d |
| --- | --- | --- | --- | --- |
| All Smoker - Non-smoker | 0.296 [0.216, 0.377] | 7.227 | 5.06×10^-13^ | 0.074 |
| Current - Non-smoker | 1.010 [0.799, 1.223] | 9.338 | 1.04×10^-20^ | 0.307 |
| Ex1 - Non-smoker | 0.706 [0.240, 1.173] | 2.966 | 3.02×10^-03^ | 0.219 |
| Ex2 - Non-smoker | 0.384 [0.276, 0.493] | 6.941 | 3.98×10^-12^ | 0.133 |
| Light1 - Non-smoker | 0.288 [-0.068, 0.645] | 1.585 | 1.13×10^-01^ | 0.100 |
| Light2 - Non-smoker | -0.011 [-0.143, 0.121] | -0.157 | 8.75×10^-01^ | 0.016 |
| Other - Non-smoker | -0.090 [-0.198, 0.019] | -1.621 | 1.05×10+ | -0.012 |

Note: As illustrated in Table S7, smokers had a bigger BrainAge Gap than non-smokers, with a mean difference (MD) of 0.296 years, p < 0.001, CI = 0.598–0.646, and Cohen’s d = 0.074. To further quantify the difference in the BrainAge Gap of smokers and non-smokers, each smoking subgroup was compared with the non-smoking group. urrent smokers had the largest BrainAge Gap, with MD = 1.101 years, p < 0.001, and Cohen’s d = 0.307, followed by the Ex-smoker1 group (MD = 0.706 years, p < 0.001, and Cohen’s d = 0.219), and Ex-smoker2 group (MD = 0.384 years, p < 0.001, and Cohen’s d = 0.133). There were no significant differences in the BrainAge Gap between the Light smoker1, Light smoker2 group and Other smoker group and the non-smokers.

## Table S9. The difference in PRS between the smoker subgroups and the non-smoker group

|  | Mean difference (CI) | t-value | p-value | Cohen’s d |
| --- | --- | --- | --- | --- |
| All Smoker - Non-smoker | 0.622 [0.598,0.646] | 50.381 | 0 | 0.633 |
| Current - Non-smoker | 1.023 [0.960, 1.086] | 31.923 | 5.2×10^-219^ | 1.138 |
| Ex1 - Non-smoker | 1.000 [0.867, 1.142] | 14.333 | 2.13×10^-46^ | 1.095 |
| Ex2 - Non-smoker | 0.941 [0.910, 0.972] | 59.230 | 0 | 0.999 |
| Light1 - Non-smoker | 0.890 [0.784, 0.995] | 16.516 | 6.11×10^-61^ | 0.969 |
| Light2 - Non-smoker | 0.851 [0.813, 0.889] | 43.890 | 0 | 0.907 |
| Other - Non-smoker | 0.057 [0.027, 0.088] | 3.652 | 2.61×10^-4^ | 0.059 |

Note: As illustrated in Table S8, smokers had a bigger PRS than non-smokers, with a mean difference (MD) of 0.622, p < 0.001, CI = 0.598–0.646, and Cohen’s d = 0.633. To further quantify the difference in the PRS of smokers and non-smokers, each smoking subgroup was compared with the non-smoking group. Current smokers had the largest PRS, with MD = 1.1023, p < 0.001, and Cohen’s d = 1.138, followed by the Ex-smoker1 group (MD = 1.000, p < 0.001, and Cohen’s d = 1.095), Ex-smoker2 group (MD = 0.941, p < 0.001, and Cohen’s d = 0.999), Light smoker1 (MD = 0.890，p < 0.001，Cohen’s d = 0.969) and Light smoker2 (MD = 0.851，p < 0.001，Cohen’s d = 0.907).

## Table S10. Sequence variants associated with Pack-year

|  | **RsNumber** | **Position (GRCh38)** | **Allele** | **MAF (%)** | **Effect(β)** | **p-value** |
| --- | --- | --- | --- | --- | --- | --- |
| **exome-wide significance** | rs199533 | chr17:46751565 | A/G | 21.25 | 0.362 | 8.825e-19 |
|  | rs7542 | chr16:30114519 | C/G | 43.98 | -0.2287 | 1.689e-11 |
| **suggestive significance** | rs864736 | chr1:214976917 | C/A | 45.19 | 0.1794 | 9.715e-08 |
|  | rs12650174 | chr4:93308358 | G/A | 7.1 | 0.3203 | 9.475e-07 |
|  | rs75298305 | chr7:131471760 | T/G | 6.919 | 0.3259 | 8.422e-07 |
|  | rs12146713 | chr12:106083027 | C/T | 9.544 | 0.2868 | 5.368e-07 |
|  | rs11618612 | chr13:109710128 | T/C | 26.1 | -0.1894 | 7.590e-07 |
|  | rs6119728 | chr20:31825318 | A/G | 29.21 | -0.196 | 1.081e-07 |

**Table S11. Association of PRS (PT=0.04) with BAG, tGMV and smoking parameter with more adjusting for sample relatedness.**

| **Group** | **Model1** | | | **Model2** | | |  |  |
| --- | --- | --- | --- | --- | --- | --- | --- | --- |
|  | β(SE) | t-value | p-value | β(SE) | t-value | p-value | |  |
|  | **BAG** | | | | | | | |
| Smoking group  (unadjusted Pack.year) | 0.09(0.032) | 2.686 | 0.0072 | 0.08(0.032) | 2.563 | 0.0104 | |  |
| Smoking group  (adjust Pack.year) | 0.04(0.049) | 0.890 | 0.374 | 0.04(0.049) | 0.785 | 0.433 | |  |
| Smoking and control group | 0.08(0.025) | 3.464 | 5.33×10^-4^ | 0.08(0.025) | 3.453 | 5.55×10^-4^ | |  |
|  | **tGMV** | | | | | | | |
| Smoking and control group  (unadjusted Pack.year) | -350.90(74.371) | -4.718 | 2.39×10^-6^ | -161.2(49.47) | -3.258 | 0.0011 | |  |
| Smoking and control group  (adjusted Pack.year) | -256.48(89.838) | -2.855 | 0.00431 | -136.11(59.777) | -2.277 | 0.0228 | |  |
|  | **Quitting duration** | | | | | | | |
| Unadjusted Pack.year | -0.76(0.179) | -4.222 | 2.46×10^-5^ | -0.75(0.179) | -4.182 | 2.94×10^-5^ | |  |
| Adjusting Pack.year | -0.31(0.151) | -2.071 | 0.0383 | -0.36(0.151) | -2.357 | 0.0185 | |  |

Note: In this association analysis, the predictor variable was PRS and response variables were shown in the table. Model 1: Adjusted for sex, age and **sample relatedness** (the first 5 principal from components genetic ancestry analysis). Model 2: Adjusted for sex, age, TIV, handedness, BMI, alcohol status, site, education and **sample relatedness** (the first 5 principal from components genetic ancestry analysis)

#
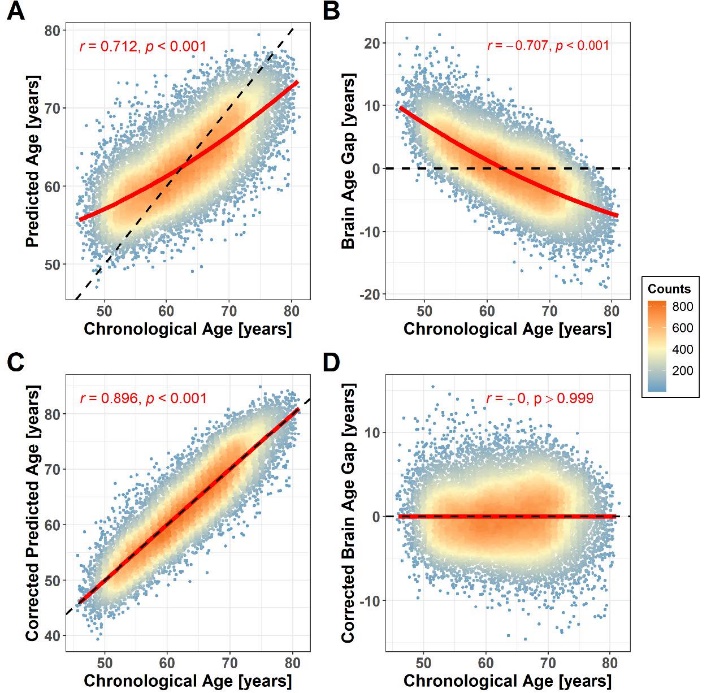
Figures

## Figure S1. The XGBoost predictor’s performance of BAG in the non-smokers

The brain age prediction models that were run on the control group using XGBoost implemented in a nested five-fold CV framework showed a prediction accuracy of r = 0.712, CI = 0.703–0.719 (Fig. A), RMSE = 5.280, and MAE = 4.220. The BrainAge Gap for every non-smokers (Fig. B) showed a negative association with chronological age, as expected (r = − 0.707). After bias adjusting using linear regression with the formula BrainAge = 51.68 + 0.151× age + 0.005 × age2, the corrected BrainAge of non-smokers was correlated more with chronological age (r = 0.896, CI = 0.893–0. 899 [Fig. C], RMSE = 3.722, and MAE = 2.997), and the corrected BrainAge Gap was orthogonal to the chronological age (r ≈ 0; Fig. D).


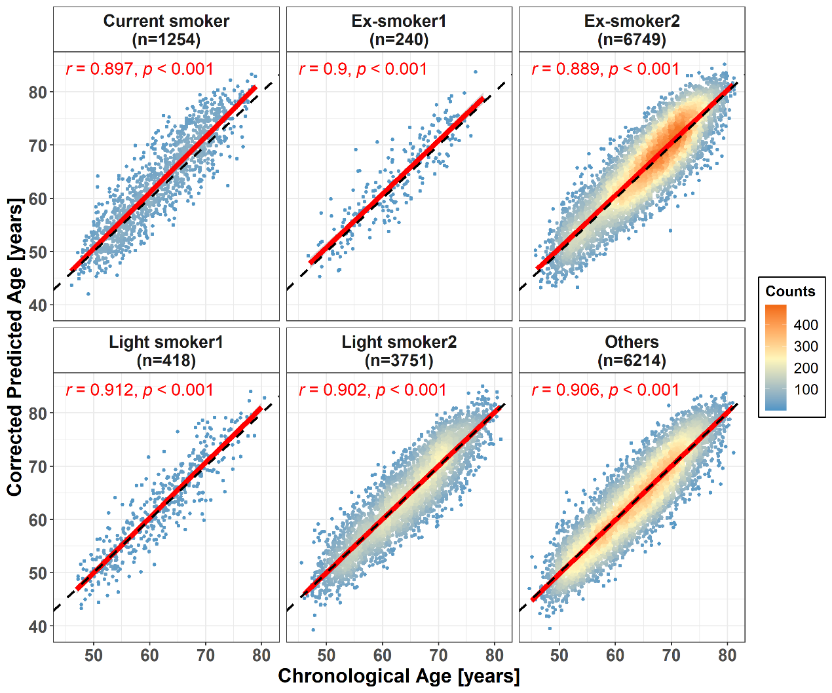
Figure S2. Correlation between the Chronological Age (X-axis) and the BrainAge (Y-axis) with age bias adjustment in all smoker subgroups.

Correlation between the corrected predicted age (i.e., BrainAge) and chronological age in the six smoking groups with r = 0.90, p < 0.001. The slope of the black dotted line is 1. The red line is the fitted curve with the linear effect of chronological age. (For interpretation of the references to color in this figure legend, the reader is referred to the web version of this article.)


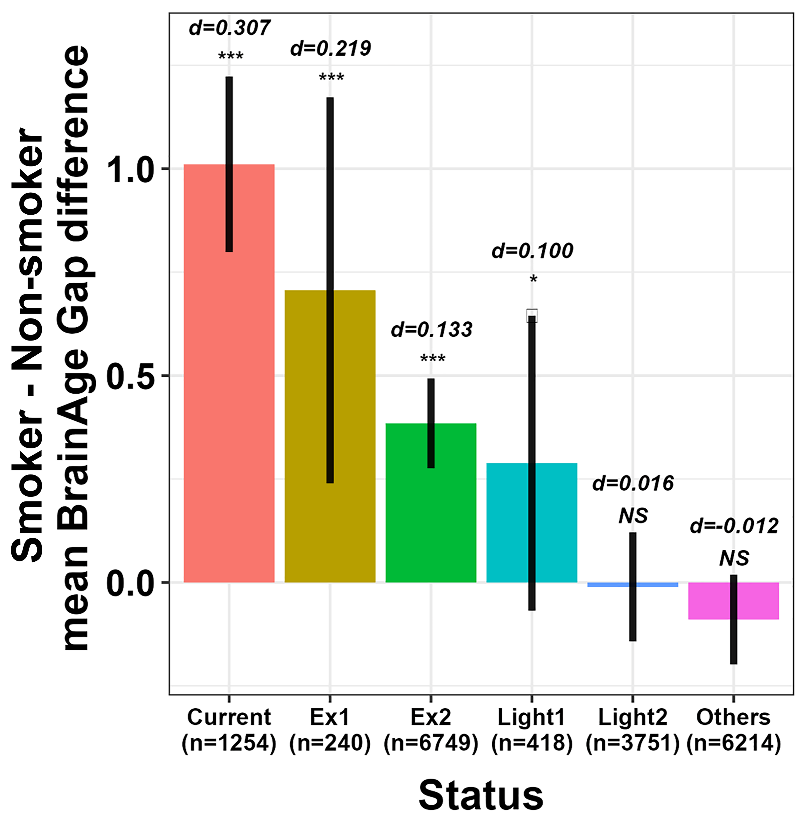
Figure S3. Difference between each smoker subgroup and non-smokers in the mean corrected BrainAge Gap. The error bar shows the 95% confidence interval. The statistical significance level is shown above each bar (****, p < 0.0001, ***, p < 0.001, **, p < 0.01, *, p < 0.5, NS, non-significant).

**
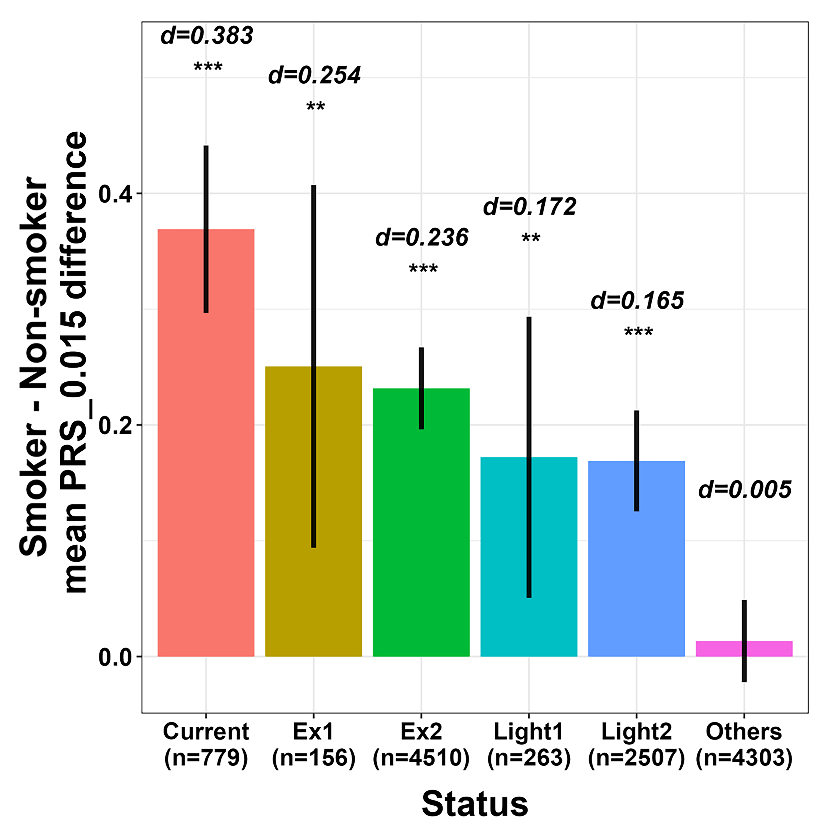
Figure S4.** Difference between each smoker subgroup and non-smokers in the PRS. The error bar shows the 95% confidence interval. The statistical significance level is shown above each bar (****, p < 0.0001, ***, p < 0.001, **, p < 0.01, *, p < 0.5, NS, non-significant).


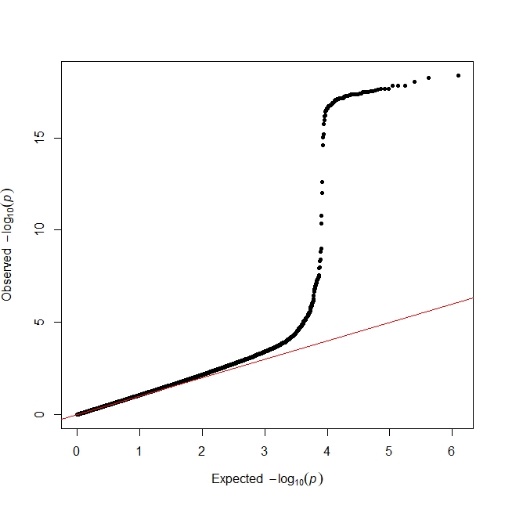


## Figure S5. The Q-Q plot of GWAS

**Figure S6. The manhattan plot of GWAS**


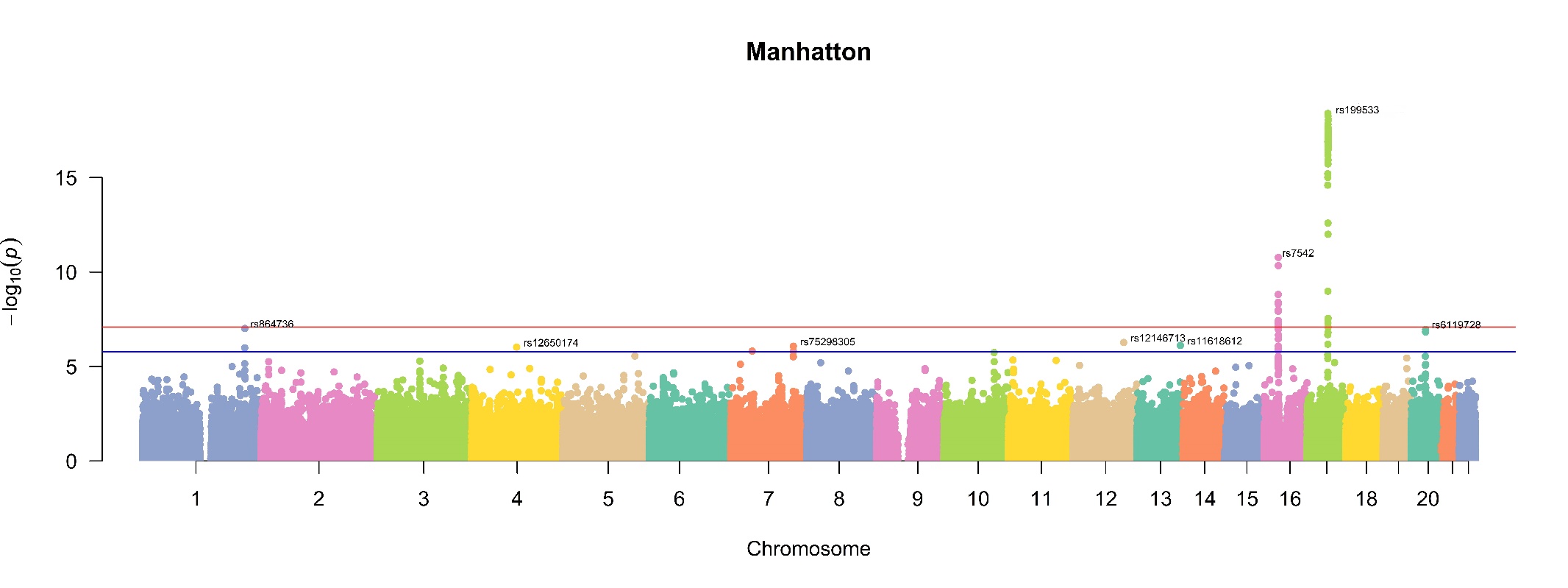


**
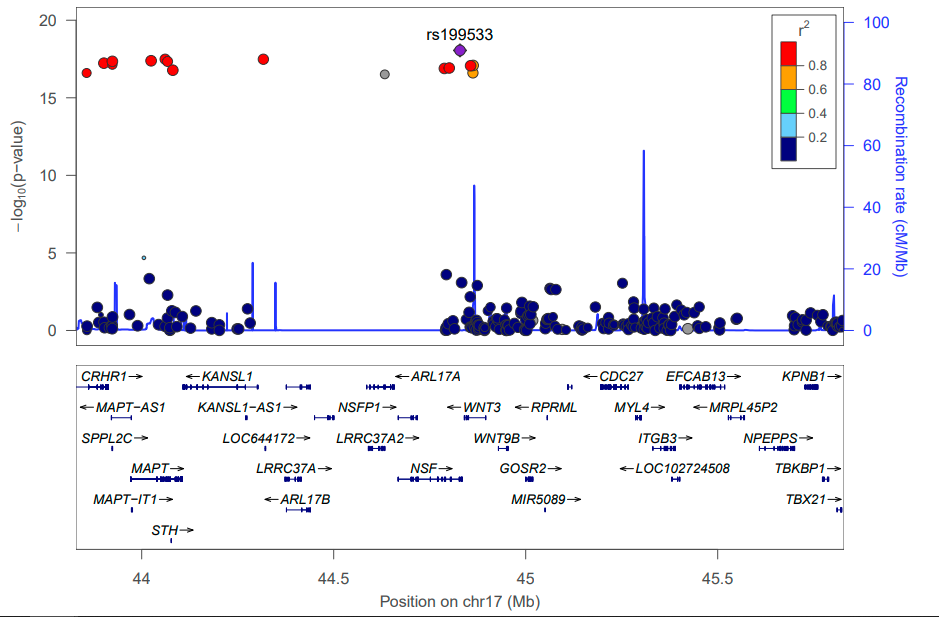
Figure S7. LocusZoom plot showing the GWAS results for the neighborhood of rs199533. r^2^: a measure of the linkage disequilibrium between rs199533 and SNPs**


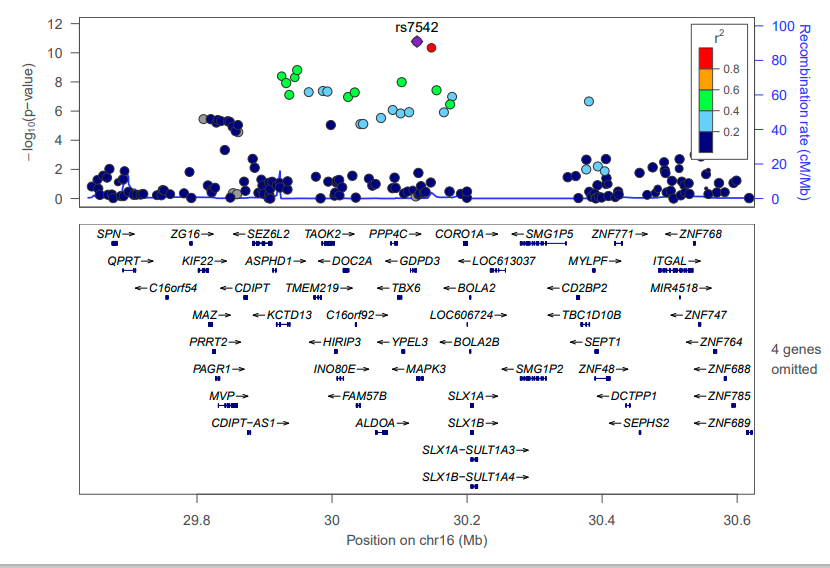
**Figure S8. LocusZoom plot showing the GWAS results for the neighborhood of rs7542. r^2^: a measure of the linkage disequilibrium between rs7542 and SNPs**


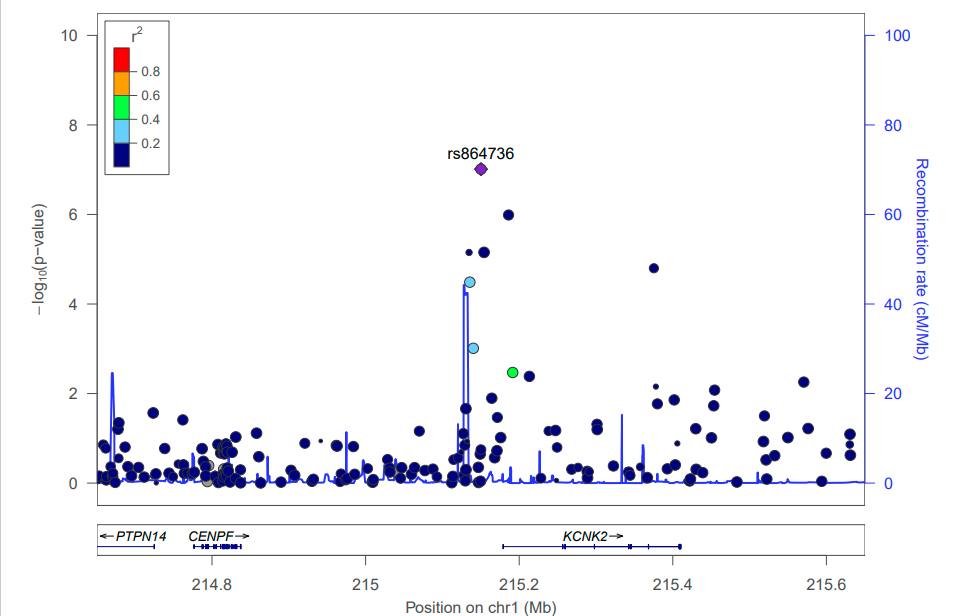
**Figure S9. LocusZoom plot showing the GWAS results for the neighborhood of rs864736. r^2^: a measure of the linkage disequilibrium between rs864736 and SNPs**


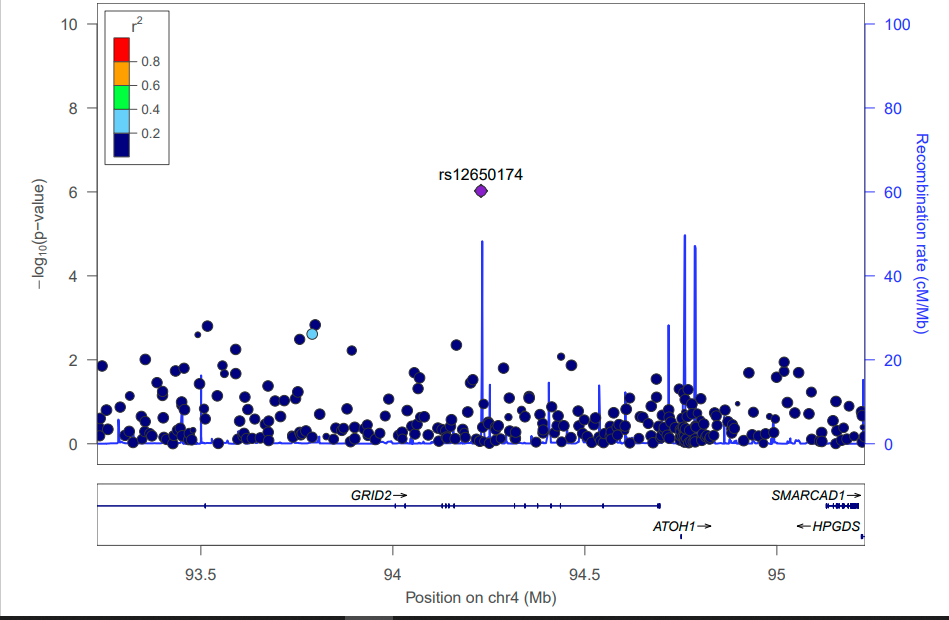
**Figure S10. LocusZoom plot showing the GWAS results for the neighborhood of rs199533. r^2^: a measure of the linkage disequilibrium between rs199533 and SNPs**


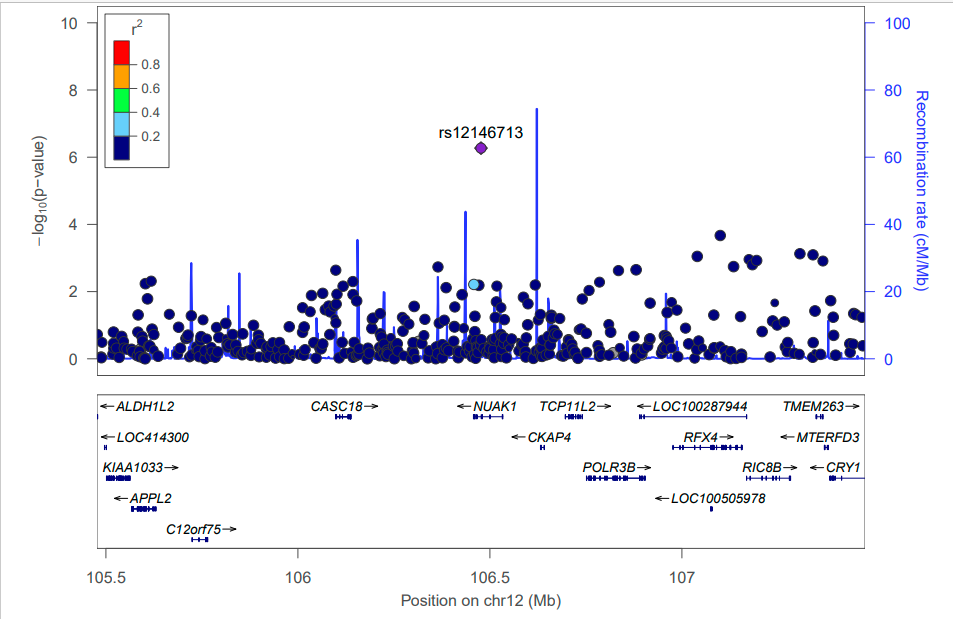


**Figure S11. LocusZoom plot showing the GWAS results for the neighborhood of rs12146713. r^2^: a measure of the linkage disequilibrium between rs12146713 and SNPs**

**
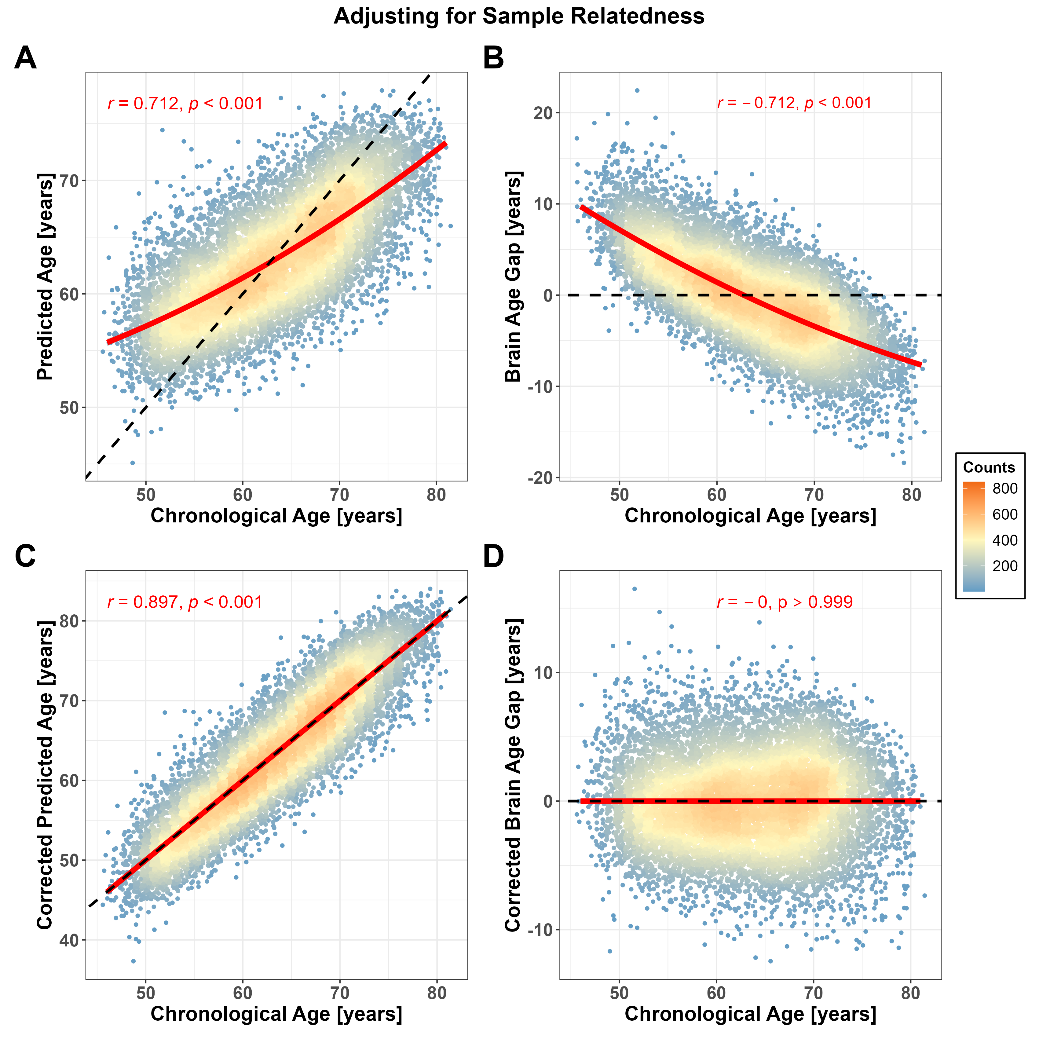
Figure S12. The XGBoost predictor’s performance of BAG in the non-smokers after adjusting for sample relatedness**

## **
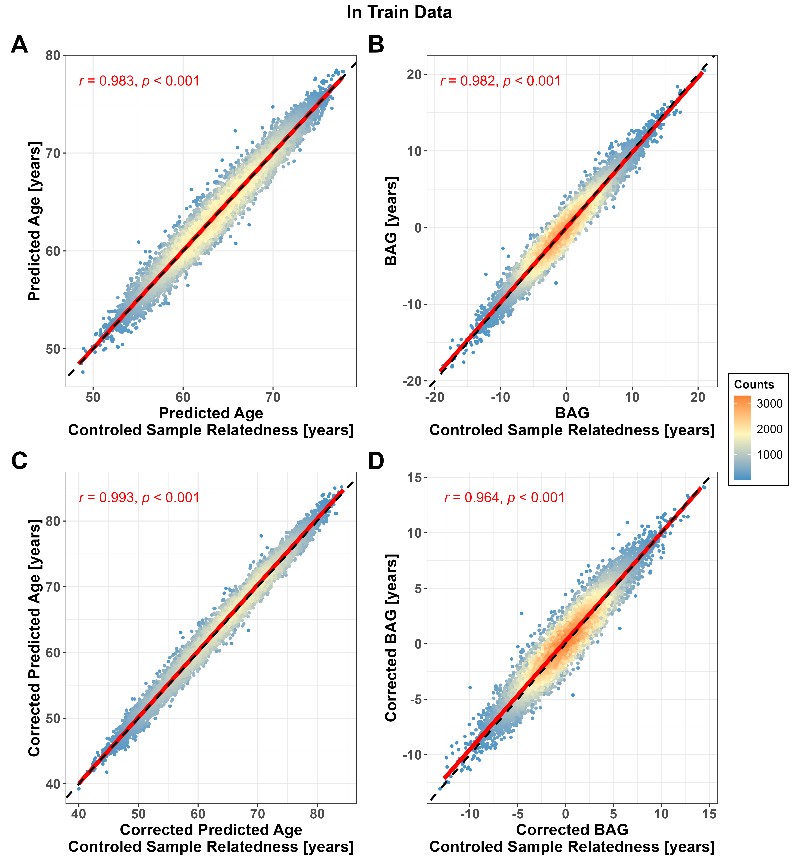
 Figure S13. Correlation between prediction results with and without adjusting** sample **correlation in train** data

## **
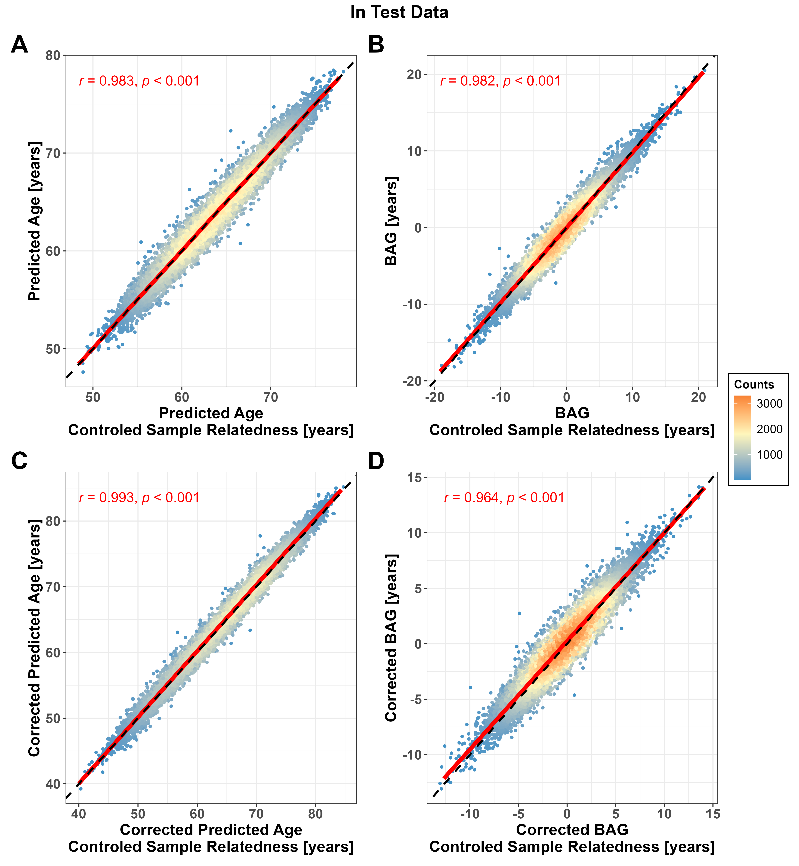
**Figure S14. Correlation between prediction results with and without adjusting sample correlation in test data
